# Mechanism of Tao Hong Decoction in the treatment of atherosclerosis based on network pharmacology and experimental validation

**DOI:** 10.3389/fcvm.2023.1111475

**Published:** 2023-01-26

**Authors:** SiJin Li, Ping Liu, Xiaoteng Feng, Min Du, Yifan Zhang, YiRu Wang, JiaRou Wang

**Affiliations:** Longhua Hospital, Shanghai University of Traditional Chinese Medicine, Shanghai, China

**Keywords:** Tao Hong Decoction (THD), atherosclerosis, network pharmacology, molecular docking, experimental validation

## Abstract

**Background:**

Atherosclerosis (AS) has long been recognized as a cardiovascular disease and stroke risk factor. A well-known traditional Chinese medicine prescription, Tao Hong decoction (THD), has been proven effective in treating AS, but its mechanism of action is still unclear.

**Objective:**

To assess the effects, explore THD’s primary mechanism for treating AS, and provide a basis for rational interpretation of its prescription compatibility.

**Methods:**

Based on network pharmacology, we evaluated the mechanism of THD on AS by data analysis, target prediction, the construction of PPI networks, and GO and KEGG analysis. AutoDockTools software to conduct Molecular docking. Then UPLC-Q-TOF-MS was used to identify significant constituents of THD. Furthermore, an AS mice model was constructed and intervened with THD. Immunofluorescence, RT-qPCR, and Western blot were used to verify the critical targets in animal experiments.

**Results:**

The network pharmacology results indicate that eight core targets and seven core active ingredients play an essential role in this process. The GO and KEGG analysis results suggested that the mechanism is mainly involved in Fluid shear stress and atherosclerosis and Lipid and atherosclerosis. The molecular docking results indicate a generally strong affinity. The animal experiment showed that THD reduced plaque area, increased plaque stability, and decreased the levels of inflammatory cytokines (NF-κB, IL-1α, TNF-α, IL-6, IL-18, IL-1β) in high-fat diet -induced ApoE-/-mice. Decreased levels of PTGS2, HIF-1α, VEGFA, VEGFC, FLT-4, and the phosphorylation of PI3K, AKT, and p38 were detected in the THD-treated group.

**Conclusion:**

THD plays a vital role in treating AS with multiple targets and pathways. Angiogenesis regulation, oxidative stress regulation, and immunity regulation consist of the crucial regulation cores in the mechanism. This study identified essential genes and pathways associated with the prognosis and pathogenesis of AS from new insights, demonstrating a feasible method for researching THD’s chemical basis and pharmacology.

## 1. Introduction

Atherosclerosis (AS) is a lipid-driven and chronic inflammatory disease. Worldwide, AS-related diseases such as heart disease and stroke are the leading cause of death (1). Various complex pathological processes are involved in the occurrence and development of AS plaques, such as lipid metabolism disorders, oxidative stress, hemodynamic changes, and inflammation (2). In recent years, low-density lipoprotein cholesterol (LDL-C) lowering regimens and therapies targeting other traditional risk factors for cardiovascular disease have substantially reduced cardiovascular mortality (3). However, despite targeted interventions, the overall benefits have stagnated, and cardiovascular disease remains a growing global burden (4).

Traditional Chinese medicine (TCM) has been widely used in China and other Asian countries for over 2,000 years (5). Through the summarization of physicians of the past dynasties, it was found that there are numerous theories concerning AS pathogenesis, including qi deficiency (Qi-xu in Chinese), blood stasis (Xue-yu in Chinese), and toxic pathogens (Du-Xie in Chinese) (6). In the early stage of AS, qi deficiency and blood stasis are the main; in the middle stage, qi and blood deficiency are the keys; and in the late stage, qi deficiency, phlegm, and blood stasis and obstruction appear. Therefore, qi and blood-activating drugs (7) are often used in clinical treatment.

Tao Hong Decoction (THD) is a classic TCM prescription that promotes blood circulation and dissipates blood stasis. THD comprises Semen Persicae (Taoren in Chinese), Flos Carthami (Honghua in Chinese), Rhizoma Ligustici (Chuanxiong in Chinese), Radix Angelicae Sinensis (Danggui in Chinese) and Radix Clematidis (Weilingxian in Chinese) (Table 1). THD is from Lei Zheng Zhi Cai, compiled by Lin Peiqin in the Qing Dynasty. Studies have shown that Taoren, Honghua, Chuanxiong, and Danggui can improve hemorheology (8), regulate the expression of inflammatory factors (9, 10), inhibit oxidative stress (11, 12), protect vascular endothelium and regulating blood lipid levels (13, 14). Accordingly, THD may be a feasible treatment considering its pharmacological mechanism of action. Nonetheless, there is still uncertainty regarding THD’s therapeutic effects and mechanisms. Due to the multi-component, multi-target, multi-effect, and overall regulatory effects of TCM, it is challenging to study herbs’ molecular mechanisms in detail. From quality control to clinical effectiveness, network pharmacology offers a new approach to studying Chinese medicine (15).

**TABLE 1:** Components of Tao Hong Decoction.

Chinese name		Botanical name	Part used	Amount (g)
Taoren	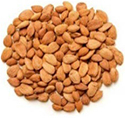	Semen Persicae	Mature seed	9
Honghua	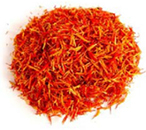	Flos Carthami	Style and stigma	9
Chuanxiong	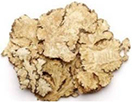	Rhizoma Ligustici	Root and stem	9
Danggui	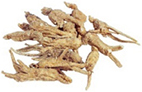	Radix Angelicae Sinensis	Root	9
Weilingxian	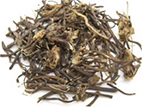	Radix Clematidis	Root and stem	9

This study used network pharmacology to predict potential targets and pathways of THD for treating AS followed by molecular docking and experimental validation. The aim is to provide a complementary therapy strategy of TCM for treating AS.

## 2. Materials and methods

### 2.1. Collection of potential active ingredients and related targets of THD

Input each medicine of THD into TCMSP data^1^ step by step (16). Oral bioavailability (OB) is determined by the same principle ≥ 30%, drug-like (DL) ≥ 0.18, which was selected to obtain the practical components and target of core drugs (17). The names of the targets mentioned above were normalized through the UniProt database^2^ (18).

### 2.2. Screening of known genes targets related to atherosclerosis

‘‘Atherosclerosis’’ was used as a keyword to screen in the DrugBank database,^3^ MalaCards database,^4^ PharmGkb database,^5^ TTD database,^6^ and GeneCards database.^7^ Meanwhile, the GeneCards database selected relevance scores greater than or equal to 1. The core targets of each database were merged, and a Venn diagram was drawn using R_v4.0.2 software.

### 2.3. Construction of the Chinese Medicines-Active Ingredients-Intersection Target network

R_v4.0.2 software was used to screen out the intersection targets of THD against AS, and the Venn diagram of the THD targets -disease targets was drawn. These targets were used to construct a “Chinese Medicines-Active Ingredients-Intersection Targets” network using Cytoscape_v3.8.0 software (19).

### 2.4. Protein-protein interaction (PPI) data

The intersection targets were input into the STRING database^8^ (20), species were set to “Homo sapiens,” and minimum required interaction scores higher than 0.400 were screened to construct the PPI network diagram. Cytoscape_v3.8.0 was used to construct the PPI network. The plug-in CytoNCA was used to analyze the network topology (21) according to Betweenness Centrality (BC), Closeness centrality (CC), Degree centrality (DC), Eigenvector Centrality (EC), Network centrality (NC), and Local edge connectivity (LC). The screening criteria are as follows: values of six parameters are all greater than the median of all nodes. The targets obtained after two consecutive screenings were regarded as the key targets.

### 2.5. GO and KEGG enrichment analyses

The GO enrichment analysis (22) as well as the KEGG pathway enrichment analysis (23) were performed by packages org.Hs.egSYMBOL2EG, colorspace, string, ggplot2, DOSE, enrichplot, cluster profile of R language (version 4.0.2). Term enrichment was measured using both *q*- and *p*-value < 0.05. In our analysis, we examined terms in the Gene Ontology (GO) categories biological processes (BP), cellular components (CC), and molecular functions (MF). To further elucidate the mechanism underlying the treatment of AS with THD, the most intersection targets associated with AS were added to the signaling pathway to identify genes related to the pathway. Finally, a diagram was drawn by packages pathview of R language.

### 2.6. Molecular docking

The PubChem database^9^ was used to retrieve the 3D structure of the small molecule ligands corresponding to the key targets and downloaded the file in the “SDF” format (24). SDF format was converted to MOL2 format using OpenBabel-3.1.1 software. To obtain PDB format files of corresponding target protein receptors, we have converted the IDS of key targets by Uniprot and then entered them into the RCSB PDB database^10^ (25). The accession numbers of UniProt and PubChem have been provided in Supplementary Tables 7, 8. PyMOL-2.4.0 was used to remove water and remove ligand molecules from receptor molecules. Hydrotreating the receptor, converting it to PDBQT format, and determining the active pocket were done with the AutoDock Tools 1.5.6 software. Finally, AutoDock Vina software was used to complete molecular docking (26). Atorvastatin, widely used in treating AS, was a positive control in this study.

### 2.7. THD preparation

Five herbs (Table 1) were in THD provided by the Pharmacy of Longhua Hospital, Shanghai University of Traditional Chinese Medicine (China). Pharmacists at Longhua Hospital identified the five species. First, the raw herbs were soaked in 10-fold water for 0.5 h, then two extractions of 1 h each were conducted. After boiling twice, the aqueous extracts were pooled, evaporated at 55 rpm (90°C, 0.8 Mpa), and consolidated to varying concentrations. Quality control measures were implemented according to Chinese State Food and Drug Administration (SFDA) guidelines.

### 2.8. UPLC-Q-TOF-MS identification of major THD constituents

Waters H-Class UPLC system (Waters Technology Co., Ltd.) coupled with an AB Sciex Triple TOF^®^ 4,600 high-resolution mass spectrometer (SCIEX company) were used for UPLC-Q-TOF-MS analyses. The UPLC separation was performed on a Waters CORTECS^®^UPLC^®^ T3 (2.1 × 100 mm, 1.6 μm). The mobile phases were acetonitrile (A) and distilled water containing 0.1% formic acid (B) at a flow rate of 0.3 ml/min. The column oven temperatures were set at 30°C, while the injection volumes for all samples were 2 μl. The mass spectrometer was operated in ESI-Negative/Positive ion mode. The Supplementary material shows gradient conditions and Mass parameters (Supplementary Tables 1, 2).

### 2.9. Animals and THD administration

Animal experimental procedures were approved by Longhua Hospital’s Animal Experimental Ethics Committee, with the record number LHERAW-22010. 6-week-old ApoE-/- male mice and C57/B6J wild-type mice (17–21 g) were purchased from GemPharmatech Co., Ltd. (Nanjing, Jiangsu, China) and bred at the specific pathogen-free (SPF) barrier system of the Animal Center of Longhua Hospital. Thirty ApoE-/- mice were randomly divided into five groups (6 mice/group): model group (MOD), low THD group (TL, 2.89 g/kg), medium THD group (TM, 5.77 g/kg), high THD group (TH, 11.54 g/kg), and Atorvastatin group (ATO, 5 mg/kg). Six C57/B6J, wild-type mice, were regarded as the control group (CON). After the 7-day adaptation period, the THD groups, model group, and Atorvastatin group were administered 12 weeks after being given the high-fat diet (HFD, 78.85% chow diet, 21% fat, and 0.15% cholesterol) for 12 weeks. We administered the THD and Atorvastatin to mice by gastric gavage. The model group received the same volume of Normal saline. Meanwhile, the control group was fed a regular diet and administered Normal saline. We monitored the health of the mice by observing changes in their weight and mental states. All mice were anesthetized with sodium pentobarbital (50 mg/kg) before obtaining the blood and tissues.

### 2.10. Evaluation of serum lipid in the mice

Blood was collected from the inner canthal region. We centrifuged blood samples at 3,000 rpm for 20 min after keeping them at room temperature for 0.5 h. A phosphate-buffered saline solution (1:2) was used to dilute the upper transparent serum. Then automatic MODULAR biochemical identification instrument was used at Longhua Hospital, Affiliated with Shanghai University of TCM, to analyze Total cholesterol (TC), triglyceride (TG), and LDL-C levels.

### 2.11. Histomorphology assay

The heart tissues were soaked in 4% paraformaldehyde (PFA) for at least 48 h. Afterward, they were dehydrated in sucrose solutions worth 10, 20, and 30% and then were prepared by removing periadventitial fat and connective tissue. An aortic root-containing third of the heart was transversally cut, embedded with OCT glue, and sliced into 10-μm-thick slices with a frozen tissue slicer. Aortic sinus sections were stained with Hematoxylin for 5 min and Eosin for 1 min to identify necrotic areas in atherosclerotic lesions. To quantify the size of lipid deposition in the aortic sinus, we have applied Oil Red O solution (0.5% in isopropanol, diluted with ddH_2_O at a 3:2 ratio) to frozen slices at 37°C for 30 min, and counterstained with Hematoxylin for 2 min.. The Masson staining of aortic sinus tissue was performed with a Trichrome stain kit (Solarbio, China) to detect collagen fibers in the plaque. Finally, all the images were analyzed and calculated by Image-Pro Plus 6.0. Data statistics were calculated using plaque area in the total aortic sinus lumen area and the percentage of positive staining across the entire plaque area.

### 2.12. Quantitative real-time PCR

According to the manufacturer’s standard protocols, aortic RNAs were extracted using RNA Purification Kit (EZBioscience, China) and reverse transcribed into cDNA using PrimeScript RT Reagent Kit (TAKARA, Japan). All the primers were synthesized by Shanghai Sangon Biotechnology (Shanghai, China). The Supplementary material lists the primer sequences (Supplementary Table 3). We conducted the Real-Time Quantitative PCR assay (RT-qPCR) with the Applied Biosystems 7500 Real-Time PCR System (Applied Biosystems, United States) using TB Green Premix EX Taq (TAKARA, Japan). Based on normalized GAPDH expression, relative mRNA expression was calculated.

### 2.13. Western blotting

The aortas were frozen in liquid nitrogen and ground into a fine powder. The total protein for each sample was extracted using RIPA lysis buffer (with 1% protease inhibitor PMSF). Bichromoninic acid (BCA) protein assay kit (Beyotime, China) was used to measure protein concentrations. Samples of proteins (45 μg) were separated by 9% SDS-page and electrophoresed at 80 V for 20 min and then separated at 200 V for 40 min (Bio-Rad, United States). The samples were then transferred to PVDF membranes (Millipore, United States) at 350 mA for 60 min. A 1 h blocking step with 5% BSA in TBST followed by overnight incubation at 4°C with primary antibodies diluted in 5% BSA in TBST. Primary antibodies used in this study were as follows: NF-κB (1:500, Santa, China), p-NF-κB (1:500, Santa, China), TNF-α(1:1,000, CST, China), IL-18(1:1,000, Abmart, China), IL-1β(1:1,000, Abmart, China), PTGS2(1:1,000, Absin, China), HIF-1α(1:1,000, Abmart, China), VEGFA (1:1,000, Abmart, China), VEGFC (1:1,000, Santa, China), FLT-4(1:1,000, Santa, China), PI3K (1:1,000, CST, China), p-PI3K (1:1,000, CST, China), AKT (1:1,000, CST, China), p-AKT (1:1,000, CST, China), P38(1:1,000, CST, China), p-P38(1:1,000, CST, China), GAPDH (1:1,000, Absin, China), α-TUBLIN (1:1,000, ABClonal, China). After washing the membranes (five times for 5 min) with TBST, the corresponding secondary antibodies were incubated at room temperature for 1 h. Following TBST rinses, enhanced chemiluminescence (ECL) kit (Epizyme, China) was used to detect protein bands. The sample was visualized and photographed using the ChemiScope 6000 imaging machine (CLINX, China). The gray-scale value of signals was determined by analyzing digital images using ImageJ Software.

### 2.14. Immunofluorescence

Aortic sinus sections were rewarmed for 10 min at room temperature. Tissues fixation with 4% paraformaldehyde for 15 min, non−specific antigen blocking with 5% BSA in PBST for 10 min, NF-κB primary antibody (1:200 dilution) incubation at 4°C overnight, goat anti-mouse IgG H&L (1:200 dilution) linking for 1 h and 4’,6-diamidino-2-phenylindole (DAPI) (Yeasen, China) counterstaining were performed. The images were captured using a fluorescence microscope and merged.

### 2.15. Statistical analysis

All experimental data were shown as mean ± SEM. Analyses of three independent experiments were performed using a one-way analysis of variance, followed by multiple comparisons based on Turkey’s method. *P <* 0.05 was considered statistically significant. GraphPad Prism 8.0. software and SPSS 26.0. were used for analysis.

## 3. Results

### 3.1. Active ingredients and targets of THD

**FIGURE 1:**
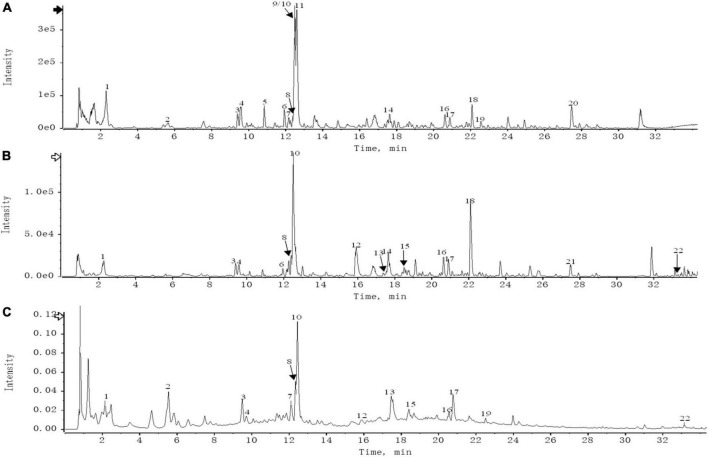
**(A)** UPLC-HRMS base peak ion current map (BPC) -negative ion mode of THD sample. **(B)** UPLC-HRMS base peak ion current map (BPC)-positive ion mode of THD sample. **(C)** UPLC UV chromatogram of THD sample-UV 254 nm.

According to the filter conditions of OB ≥ 30% and DL ≥ 0.18, the compounds of THD in Chuanxiong, Danggui, Honghua, Taoren, and Weilingxian were 7, 2, 30, 23, and 7, respectively. The TCMSP databases were searched to acquire 421 targets for the potential active compounds, and the numbers of potential targets for Chuanxiong, Danggui, Honghua, Taoren, and Weilingxian were 42, 69, 100, 139, and 71, respectively. After merging and removing duplicates, 92 valid targets and 40 active compounds in five traditional Chinese medicines were identified from the TCMSP database in THD. As a result of UPLC-QTOF-MS analysis, 22 primary components of THD were identified (Figure 1 and Supplementary Table 4).

### 3.2. Acquisition of THD and AS intersection targets

**FIGURE 2:**
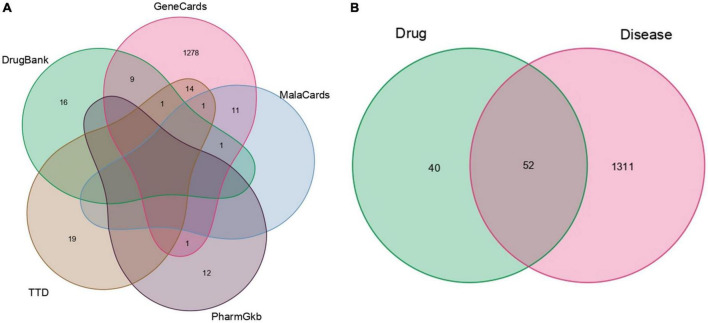
Venn diagrams. **(A)** AS-related genes. **(B)** THD targets and AS targets.

We retrieved 1316, 13, 13, 35, and 27 targets from Gencards, Malacards, PharmGKb, TTD, and Drugbank databases, respectively. A total of 1,363 important targets were obtained after merging and removing duplicates. The potential targets of THD were mapped to the union targets of AS, and 52 intersection targets were identified (Figure 2).

### 3.3. Construction of the Chinese Medicines-Active Ingredients-Intersection Target network

**FIGURE 3:**
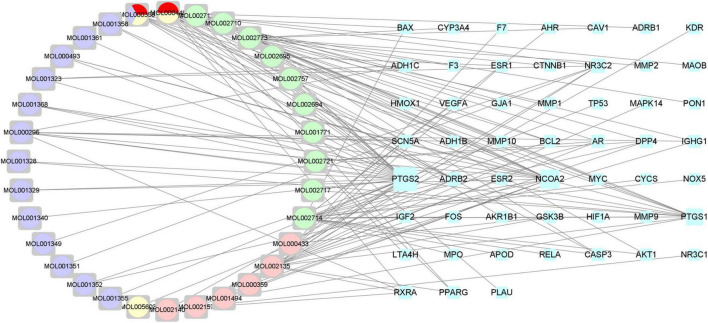
Chinese medicines-active ingredients-intersection targets network. The blue nodes on the right represent the intersection targets. The round nodes on the left represent all the herbs of THD. The pink nodes represent Rhizoma Ligustici. The green nodes represent Flos Carthami. The red nodes represent Radix Angelicae Sinensis. The yellow nodes represent Radix Clematis. The purple nodes represent Semen Persicae.

Through the precise matching of compounds with intersection targets, a total of 32 active THD ingredients for treating AS were derived (Supplementary Table 5). The Chinese Medicines-Active Ingredients-Intersection Target network diagram was constructed based on the interactions among the five herbs, 32 active compounds, and 52 targets associated with AS (Figure 3). Eighty-four network nodes represent active compounds and targets, and 139 edges show their interactions. The degree represents the number of edges associated with the node. The higher the degree, the more targets there are. Based on results from a topological analysis, the top 7 core active ingredients shown in Table 2 play a crucial role in treating AS.

**TABLE 2:** The top seven core active ingredients of THD for the treatment of AS.

Herb name	Molecule ID	Molecule name	OB (%)	DL	Degree
Flos Carthami (Honghua)	MOL002714	Baicalein	33.52	0.21	21
Flos Carthami (Honghua)	MOL002773	Beta-carotene	37.18	0.58	15
Rhizoma Ligustici (Chuanxiong)	MOL002135	Myricanone	40.6	0.51	15
Radix Angelicae Sinensis (Danggui)	MOL000449	Stigmasterol	43.83	0.76	14
Radix Clematidis (Weilingxian)	MOL000358	Beta-sitosterol	36.91	0.75	9
Semen Persicae (Taoren)	MOL000296	Hederagenin	36.91	0.75	8
Flos Carthami (Honghua)	MOL002712	6-Hydroxykaempferol	62.13	0.27	6

### 3.4. Construction of PPI and core targets network

**FIGURE 4:**
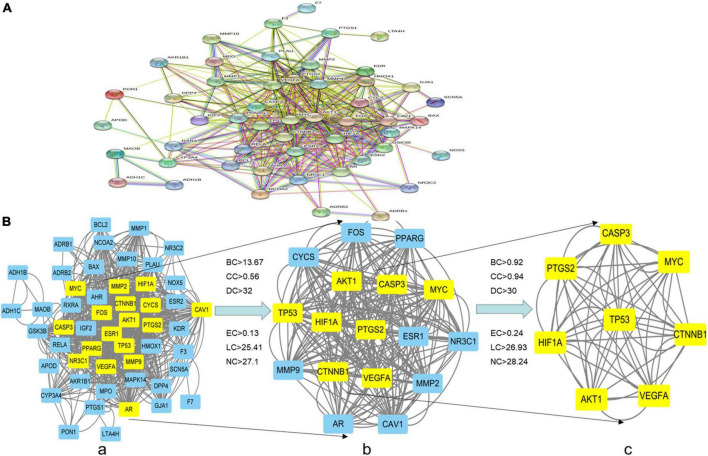
PPI network of the intersection targets of THD against AS. **(A,B)** The network topology analysis of the PPI network. **(a)** PPI network of the intersection targets, with 52 nodes and 834 edges. **(b)** The subnetwork, after first filtering, comprises 17 nodes and 258 edges. **(c)** After the second filtration, the core network comprises eight nodes and 56 edges.

After the 52 intersection targets were added to the string database, the PPI network map was generated (Figure 4A). Targets are represented as nodes interconnected by edges in a PPI network. The denser the edges of critical targets are, the bigger the nodes are, and the more significant their role in the PPI network. We screened the core targets using the CytoNCA plug-in of Cytoscape 3.8.0 and the subnetwork of 8 core targets (MYC, TP53, PTGS2, VEGFA, HIF-1α, CASP3, AKT1, CTNNB1) was obtained after two filters with scores higher than the median value of BC, CC, DC, EC, LC, and NC (Figure 4B).

### 3.5. Gene ontology analysis

**FIGURE 5:**
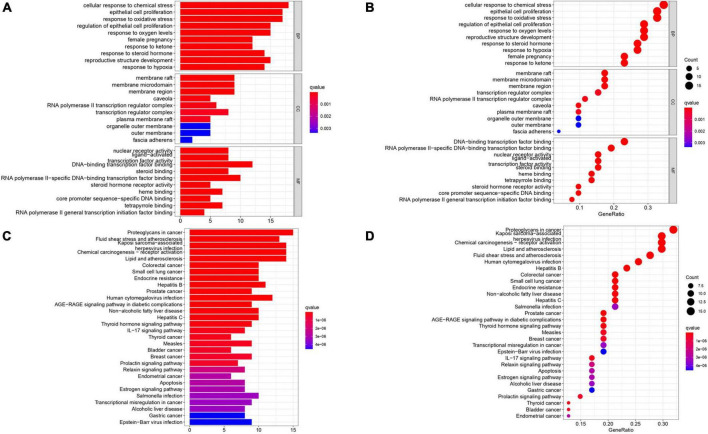
Gene Ontology terms and KEGG pathway enrichment of 52 intersection targets. **(A,B)** The top 10 GO functional terms were selected (*P <* 0.05). The spot size represented a count of genes, and color represented the *q*-value. **(C,D)** The top 30 pathways were selected (*P <* 0.05). The color represents the *q*-value, and the abscissa represents the number of genes.

1839 GO terms were identified by GO enrichment analysis of 52 intersection targets related to AS. The top 10 representative clusters of BP, CC, and MF were screened according to *P*-value < 0.05, shown as bubble charts (Figure 5A). We can find that the top three BP were a cellular response to chemical stress (18 genes enriched), epithelial cell proliferation (17 genes enriched), and response to oxidative stress (17 genes enriched). The top three CC were the membrane raft (9 genes enriched), membrane microdomain (9 genes enriched), and membrane region (9 genes enriched). The top three MF were DNA-binding transcription factor binding (12 genes enriched), RNA polymerase II-specific DNA-binding transcription factor binding (10 genes enriched), and nuclear receptor activity (8 genes enriched).

### 3.6. KEGG pathway enrichment analysis

**FIGURE 6:**
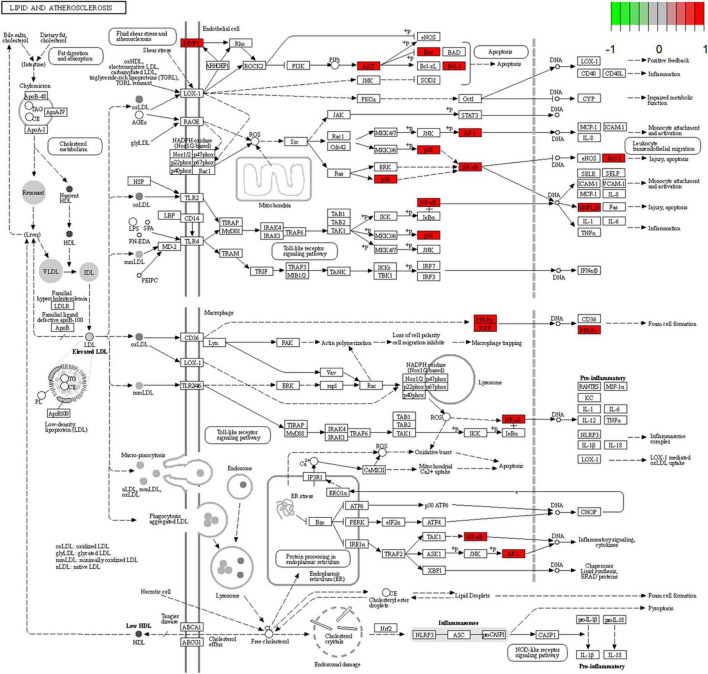
Lipid and atherosclerosis signaling pathway. Red represents the intersection targets of THD against AS in the signaling pathway.

**FIGURE 7:**
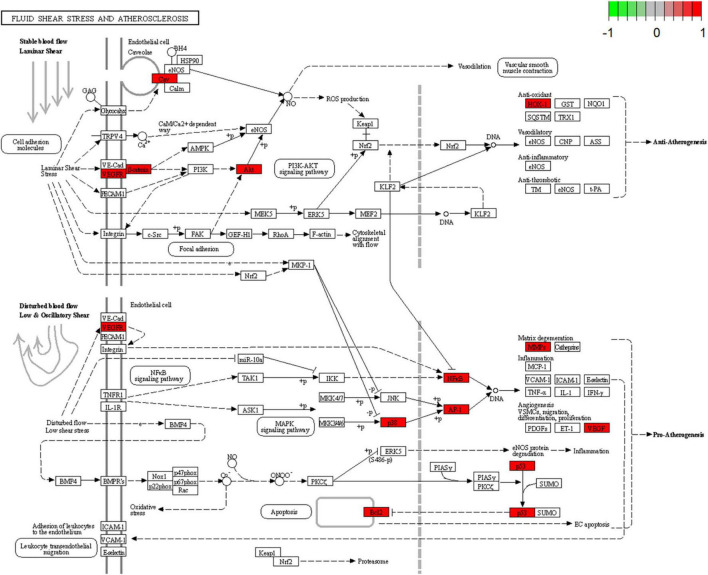
Fluid shear stress and atherosclerosis signaling pathway. Red represents the intersection targets of THD against AS in the signaling pathway.

A total of 52 THD predicted targets associated with AS were imported into the R 4.0.2 software, and the 126 signaling pathways were obtained. We selected the top 30 most enriched entries to make barplot charts (Figure 5B). The results showed that many targets were mainly enriched in the Proteoglycans in cancer (15 genes enriched), Lipid and atherosclerosis (14 genes enriched), Fluid shear stress and atherosclerosis (13 genes enriched), and Chemical carcinogenesis—receptor activation (14 genes enriched). Additionally, two signaling pathways with the most intersection targets associated with AS were plotted, respectively (Figures 6, 7).

### 3.7. Validation of molecular docking

**FIGURE 8:**
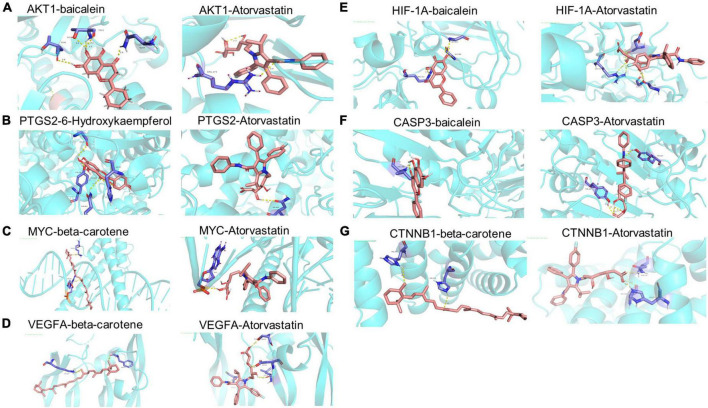
Molecular dockings of the 8 core targets and their corresponding ingredients. **(A)** AKT1 and baicalein (−10.1 kcal.Mol), AKT1 and Atorvastatin (−10.2 kcal.Mol). **(B)** PTGS2 and 6-Hydroxykaempferol (−9.3 kcal.Mol), PTGS2 and Atorvastatin (−8.5 kcal.Mol). **(C)** MYC and beta-carotene (−7.8 kcal.Mol), MYC and Atorvastatin (−8.4 kcal.Mol). **(D)** VEGFA and beta-carotene (−7.9 kcal.Mol), VEGFA and Atorvastatin (−6.7 kcal.Mol). **(E)** HIF-1A and baicalein (−8.6 kcal.Mol), HIF-1A and Atorvastatin (−7.2 kcal.Mol). **(F)** CASP3 and baicalein (−7.5 kcal.Mol), CASP3 and Atorvastatin (−8.7 kcal.Mol). **(G)** CTNNB1 and beta-carotene (−6.6 kcal.Mol), CTNNB1 and Atorvastatin (−6.6 kcal.Mol).

To investigate the binding activity of the main active ingredients of THD, we performed docking simulations of the eight core targets in the network and their corresponding core ingredients, respectively. Atorvastatin, widely used in treating AS, was used as a control (Figures 8A–G and Supplementary Table 6). Generally, binding energy ≤ −5 kcal/mol indicates that the receptors and ligands have decent binding properties. The lower the binding energy between molecules, the better the docking effect will be (27). From Supplementary Table 6, Our results showed that the binding energies of all the core targets and their corresponding ingredients were ≤ −5 kcal/mol, and the docking score was close to that of the positive control drug atorvastatin, indicating a generally strong affinity. AKT1 and baicalein showed the best binding ability, with the lowest binding energy = −10.1 kcal/mol. As displayed in Figure 8A, the structure of baicalein could form bonds with T291, T211, and N204 in AKT1. In Figure 8B, the structure of 6-Hydroxykaempferol could generate bonds with GLY-45, TYR-130, HIS-39, and GLN-461 in PTGS2. As illustrated in Figure 8C, the structure of beta-carotene could generate bonds with LYS-42 and DG-15 in MYC. As displayed in Figure 8D, the structure of beta-carotene could form bonds with LYS-107 and PHE-47 in VEGFA, respectively. Also, in Figure 8E, bonds were generated to bind baicalein with ARG-312, GLY-253, and GLY-309 in HIF-1A. Moreover, baicalein interacted with VAL-266 in CASP3 (Figure 8F). As we can see in Figure 8G, beta-carotene changes the structure of CTNNB1 by interacting with HIS-219 and HIS-260. In conclusion, an interplay exists between core targets and active ingredients of THD through different bonds.

### 3.8. THD improved the AS mice phenotype

**FIGURE 9:**
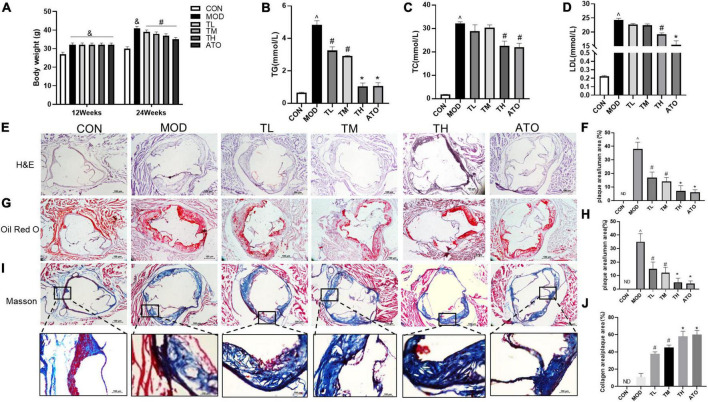
THD improved the AS mice phenotype. **(A)** THD attenuates body weight in mice. **(B–D)** Effect of THD on blood lipids in mice. H&E staining **(E)** and Oil Red O staining **(G)** were used to visualize lipid-rich plaque progression. Scale bar = 100 μm (40×). **(I)** The collagen content was determined by Masson staining. Scale bar = 100 μm (40×, Enlarged 200×). The areas of the lesion **(F–H)** and collagen content **(J)** were calculated, respectively, by Image J. Data are presented as the mean ± SEM (*n* = 3). ^∧^*P <* 0.01 vs. CON group; ^#^*P <* 0.05, *P < 0.01 vs. MOD group. ND, not detected.

After 12 weeks of HFD, the mice in the model, THD, and ATO groups weighed significantly more than those in the CON group (*P* < 0.05), while THD or ATO-treated mice had lower body weights than the control group after 12 weeks of intervention (*P* < 0.05, Figure 9A).

TG, TC, and LDL-C levels were significantly higher in the MOD group than in the CON group. In contrast, all three indices decreased substantially following THD or ATO interventions for 12 weeks (*P* < 0.05, Figures 9B–D).

To investigate the effect of THD on atherosclerotic lesions in the aortic sinus of mice, lipid plaques were observed in the aorta using H&E and Oil Red O staining. As shown in Figures 9E–H), no evidence of lipid accumulation had been detected in the CON group (*P* < 0.01), but it was significantly greater in the MOD group. In contrast, in groups treated with THD or ATO, atherosclerotic lesions showed significant reductions (*P* < 0.01) compared to MOD groups. Based on the above results, THD could reduce AS plaques in HFD-induced- ApoE-/- mice.

The characteristics of a vulnerable plaque include a low fibrous cap area (28, 29). Then the collagen content was detected by Masson staining to explore the effect of THD on the stability of atherosclerotic plaques. Figures 9I, J showed that the atherosclerotic lesions of the MOD group had a lower collagen content than the CON group (*P* < 0.01). THD and ATO increased collagen content (*P* < 0.05). According to these results, THD could improve the stability of atherosclerotic plaque, reducing its rupture risk.

### 3.9. THD decreases inflammatory cytokine levels in AS mice

**FIGURE 10:**
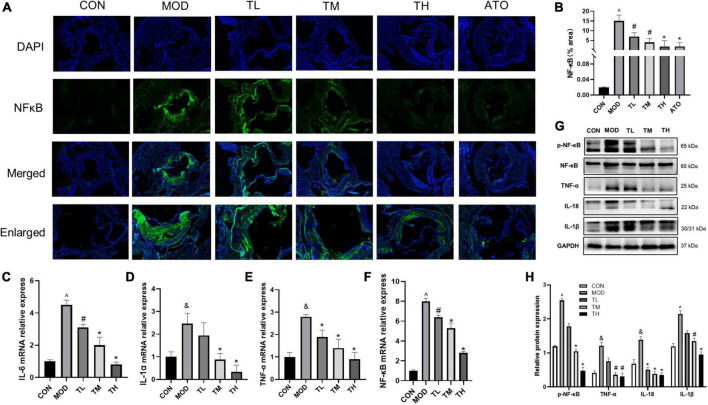
Effects of THD on inflammatory cytokines in AS Mice. **(A)** Representative photomicrographs of atherosclerotic plaque stained with NF-κB in the aortic root. Scale bar = 100 μm (40×, Enlarged 200×). **(B)** The NF-κB fluorescence area and intensity were quantized as a percentage, *n* = 3. **(C–F)** Aorta gene expression of IL-6, IL-1α, TNF-α, and NF-κB was detected by RT-qPCR, *n* = 3. **(G)** Protein expression of NF-κB, TNF-α, IL-18, and IL-1β in the aortas was investigated by Western blot. **(H)** The quantitative results of the western blot were depicted. MOD group was compared to the CON group, and THD was compared with the MOD group. GAPDH was used as an internal reference. Data are expressed as mean ± SEM. ^&^*P* < 0.05, ^∧^*P <* 0.01 vs. CON group; ^#^*P <* 0.05, *P < 0.01 vs. MOD group.

To explore the effect of THD on inflammation, we have performed immunofluorescence staining of NF-κB. As shown in Figures 10A, B, NF-κB expression was markedly increased in the atherosclerotic lesions of the MOD group (*P <* 0.01). THD and ATO down-regulated the increase of NF-κB (*P <* 0.05). Additionally, we detected inflammatory cytokines’ gene and protein expression levels in the aortas. As shown in Figures 10C–H, the MOD group had significantly up-regulated mRNA expression of IL-6, IL-1α, TNF-α, NF-κB and protein expression of NF-κB, TNF-α, IL-18, IL-1β in comparison to the CON group. It was restored after THD administration (*P <* 0.05).

### 3.10. Effects of THD on prediction targets and pathways in AS mice

**FIGURE 11:**
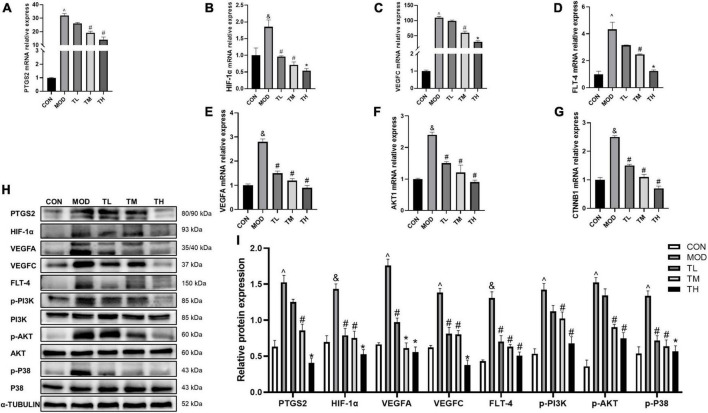
Effects of THD on prediction targets and pathways in AS Mice. **(A–G)** Aorta gene expression of PTGS2, HIF-1α, VEGFA, VEGFC, FLT-4, AKT1, CTNNB1 were detected by RT-qPCR, *n* = 3. **(H)** Protein expression of PTGS2, HIF-1α, VEGFA, VEGFC, FLT-4, PI3K, p-PI3K, AKT, p-AKT, P38, and p-P38 in the aortas were investigated by Western blot, *n* = 3. **(I)** The quantitative results of the western blot were depicted. MOD group was compared to the CON group, and THD was compared with the MOD group. α-TUBLIN was used as an internal reference. Data are expressed as mean ± SEM. ^&^*P <* 0.05, ^∧^*P <* 0.01 vs. CON group; ^#^*P <* 0.05, *P < 0.01 vs. MOD group.

To Determine whether the beneficial effect of THD on aortic roots is related to the prediction targets and signaling pathways detected by network pharmacology. we examined the mRNA expression of PTGS2, HIF-1α, VEGFA, VEGFC, FLT-4, AKT1, CTNNB1 and the protein expression of PTGS2, HIF-1α, VEGFA, VEGFC, FLT-4, PI3K, AKT, and P38 in the aortas. Furthermore, phosphorylation of P38, PI3K, and AKT was examined by Western Blot assays. Compared to the CON group, the RT-qPCR and Western blotting showed that the mRNA (Figures 11A–G) and protein expression (Figures 11H, I) in the MOD group was significantly up-regulated (*P <* 0.05) and restored after THD administration (*P <* 0.05).

## 4. Discussion

The occurrence and development of atherosclerotic diseases may result from a combination of factors such as lipid accumulations and inflammatory immune cells in the blood vessel intima (30). Complex diseases may have a polygenic or even “omnigenic” etiology (31). Consequently, multi-target combinational therapeutic strategies have become increasingly important. The traditional Chinese herbal mixture combines multiple herbs, multibioactive components, and multiple targets and pathways, making it ideal for treating complex diseases (32). THD is a traditional Chinese herb mixture used for centuries to treat cardiovascular diseases and has good effectiveness. Several studies have shown that THD can reduce inflammation, protect nerves, and lower levels of the N-terminal brain natriuretic peptide (NT-proBNP) in patients with chronic heart failure (33–35). Hydroxysafflor and tetramethylpyrazine are two of the main components of THD. Our previous studies suggest that it can prevent atherosclerosis through anti-inflammatory action, ameliorating lipid metabolism disorder, anti-oxidant action, and inhibition of angiogenesis (36, 37).

### 4.1. Summary of main results

THD contained 69 active ingredients in this study, and 421 potential targets were obtained. There are 52 shared targets between THD and AS. A total of 32 active THD ingredients for treating AS were derived. UPLC-QTOF-MS identified Twenty-two primary components of THD. After topological analysis, we have obtained the top 7 core active ingredients and eight-core targets. The results of GO enrichment analysis mainly cluster on cellular response to chemical stress, membrane raft, and DNA-binding transcription factor binding. The results of the KEGG analysis suggested that the mechanism is primarily involved in Fluid shear stress and atherosclerosis, Lipid, and atherosclerosis. According to the docking data, some target-compound pairs, such as PTGS2 and AKT1, had good docking affinity, and the docking score was close to that of the positive control drug atorvastatin. Thus, some of the protective effects of THD in AS may be achieved by regulating these genes. Animal experiments showed that THD regulated lipid metabolism, reduced plaque area, and increased plaque stability. By testing the above indicators, we verified that angiogenesis regulation, oxidative stress regulation, and immunity regulation are the crucial regulation cores in the mechanism.

### 4.2. Analysis of active ingredients

The primary active ingredients (baicalein, beta-carotene, myricanone, stigmasterol, beta-sitosterol, hederagenin, and 6-HydroxyKaempferol) of THD for AS treatment were identified. As a natural phenolic anti-oxidant and anti-atherosclerotic agent, baicalein modulates nitric oxide release under OX-LDL conditions (38). Meanwhile, a study of baicalein’s benefits on vascular diseases demonstrated attenuation of ICAM-1 in cultured human endothelial cells induced by IL-1β and TNFα (39). As a source of Vitamin A, beta-carotene can regulate lipid metabolism to prevent atherogenesis in ApoE-/- mice (40). Myricanone is a flavonoid that triggers caspase activation and suppresses cell proliferation by downregulating NF-κB and STAT3 signal chains (41). The phytosterol stigmasterol is classified as a tetracyclic triterpene. Its inflammation was reduced by releasing anti-inflammatory cytokines, reducing inflammatory mediator release, and inhibiting iNOS and COX2 (42). It has been demonstrated by several *in vitro* studies that beta-sitosterol possesses anti-inflammatory properties. Beta-sitosterol has antiatherogenic potential through its anti-inflammatory action on the endothelial cells of human aortic vessels (43).

Moreover, the modulation of NF-κB by beta-sitosterol has altered the inflammatory response in a rat model of sepsis (44). According to the report, hederagenin has anti-inflammatory activities (45). The anti-inflammatory effect of hederagenin reduced inflammation, inhibited phosphorylation of p38 MAPK, and decreased the production of proteins related to dorsal root ganglions, which relieved sciatica symptoms (46). The 6-hydroxykaempferol 3,6-di-O-glucoside-7-O-glucuronide (HGG) is classified as flavanol, a representative 6-hydroxykaempferol glycoside in Safflower. HGG can protect against endothelial dysfunction and prevent thrombosis by regulating the expression of HIF-1α and NF-κB (47). Overall, these seven core compounds regulate lipids and immune inflammation. Thus the core compounds of THD may act on atherosclerosis through these pathways.

### 4.3. Analysis of potential targets

The Chinese Medicines-Active Ingredients-Intersection Target network showed that the seven core compounds interacted with various targets, indicating their comprehensive and complex roles in treating AS. Coincidentally, eight targets, namely MYC, TP53, PTGS2, VEGFA, HIF-1α, CASP3, AKT1, and CTNNB1, have been identified as core genes in the PPI network of AS target proteins. PTGS2, a potent enzyme in prostaglandin biosynthesis, contributes significantly to the inflammatory response (48). Prior study has noted that PTGS2 may be associated with AS (49). VEGFA was formerly considered a proinflammatory biomarker in endothelial cells involved in AS (50). Furthermore, It plays a crucial role in pathological and developmental angiogenesis (51). HIF-1α was closely associated with fibrosis of tissues and intracellular oxygen metabolism (52). Macrophage HIF-1α promotes lipid uptake and proinflammatory responses contributing to AS (53). HIF-1α also responsible for initiating the transition from endothelial to mesenchymal cells, which leads to an increase in inflammation and proliferation, which may also contribute to the development of AS (54). AKT1 is a significant regulator of endothelial NO synthase activity and plays a role in atherosclerosis, inflammation, and angiogenesis (55). The gene expression of PTGS2, VEGFA, HIF-1α, AKT1, and CTNNB1 were found to be up-regulated in the aortas of AS mice (*P* < 0.05), and the molecular docking results indicated a generally strong affinity. It means that the protective effects of THD in AS may be achieved by regulating the targets listed above.

### 4.4. Possible cellular and molecular mechanisms of THD against AS

As determined by GO analysis, further investigation of the mechanism of THD revealed that it is involved in biological processes closely related to AS, including cellular response to chemical stress, epithelial cell proliferation, and response to oxidative stress, which promote the mechanism explaining of THD in treating AS. The results of the KEGG pathway analysis suggest THD is mainly involved in the Lipid and atherosclerosis and Fluid shear stress and atherosclerosis signaling pathways. Nevertheless, numerous pathways, such as PI3K/AKT, P38 MAPK, and VEGF signaling pathways, cooperatively correlated with the process of AS, could be found in the enrichment analysis results.

PI3K/AKT signaling pathway affects many biological processes, including cell survival, inflammation, and apoptosis (56, 57). According to studies, the PI3K/Akt signaling pathway participates in hypoxia-ischemia and regulates the expression of HIF-1α (58). HIF-1α can further regulate downstream proteins involved in angiogenesis, such as VEGF, to facilitate ischemic adaptation (59). Additionally, VEGF may contribute to plaque formation and destabilization during atherosclerosis by causing plaque ruptures (60). Five mammalian factors comprise the VEGF family: VEGFA, VEGFB, VEGFC, VEGF-D, and placenta growth factor (PlGF). In mice, VEGFA and VEGFC contribute to the development of blood vessels and lymphatic vessels, respectively. The VEGFC binds to tyrosine kinase receptors VEGFR-3 (also known as FLT-4), primarily expressed on lymphatic endothelial cells (61). The VEGF family members VEGFA and VEGFC are often overexpressed in AS. P38 MAPK is a classic signaling pathway in mammals. The role of P38 MAPK in inflammation and atherosclerosis has been implicated in several studies (62). It has been demonstrated that phosphorylated P38 MAPK can regulate inflammation through the NF-κB signaling pathway (63). NF-κB regulates PTGS2 and other cytokines to trigger the inflammatory response (64).

**FIGURE 12:**
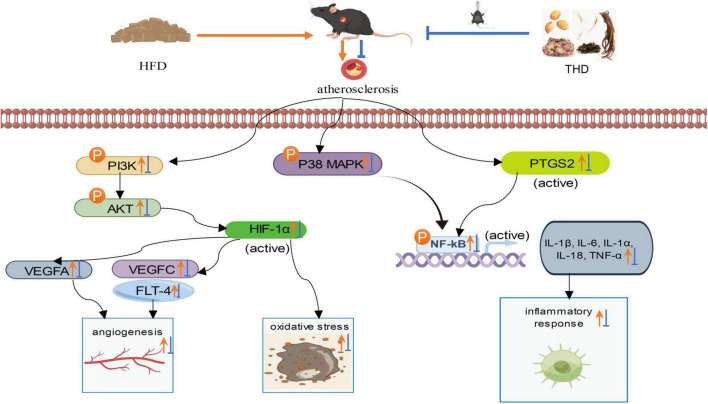
Protective mechanism of THD against HFD-induced AS mice.

In this study, animal experiments were performed to verify the expression of VEGFA, VEGFC, FLT-4, PTGS2, and HIF-1α. The phosphorylation of PI3K, AKT, and P38 and the expression of inflammatory cytokines (NF-κB, IL-1β, IL-6, IL-1α, IL-18, TNF-α) were also detected. These results suggested THD could mitigate the activation of PI3K/AKT/HIF-1α/VEGF, P38 MAPK, and PTGS2/NF-κB signaling pathways in AS mice, demonstrating that THD can intervene in atherosclerosis by regulating angiogenesis and oxidative stress, and inhibiting inflammatory response (Figure 12).

### 4.5. Limitation

However, this study had some limitations. Firstly, due to technical limitations, UPLC-Q-TOF-MS did not retrieve all the active ingredients of THD. Secondly, the current study needs to consider interactions between components and the content of each component. It would be ideal for studying in greater depth the active components, absorption mechanisms, and metabolic forms of bioactive components of THD.

## 5. Conclusion

In summary, this study preliminarily revealed the pharmacological effects of THD on AS. THD may achieve the treatment of AS by regulating angiogenesis and oxidative stress and inhibiting the inflammatory response. This study lays the foundation for further research on THD’s mechanism in treating AS.

## Data availability statement

The original contributions presented in this study are included in the article/Supplementary material, further inquiries can be directed to the corresponding author.

## Ethics statement

The animal study was reviewed and approved by the animal experimental procedures were approved by Longhua Hospital’s Animal Experimental Ethics Committee, with the record number LHERAW-22010.

## Author contributions

SL was a major contributor to writing the manuscript. PL conceived and designed the experiments, reviewed, and edited the final manuscript. SL and XF performed the experiments. MD, YZ, YW, and JW analyzed the results. All authors contributed to the article and approved the submitted version.

## Footnotes

1
https://old.tcmsp-e.com/tcmsp.php

2
https://www.uniprot.org/

3
https://go.drugbank.com/

4
https://www.malacards.org/

5
https://www.pharmgkb.org/

6
http://db.idrblab.net/ttd/

7
http://www.genecards.org/

8
https://string-db.org/

9
https://pubchem.ncbi.nlm.nih.gov/

10
http://www.rcsb.org/
