# Supplementary figures and images for: Mechanism of Tao Hong Decoction in the treatment of atherosclerosis based on network pharmacology and experimental validation
Source: Front Cardiovasc Med. 2023 Jan 26;10:1111475. doi: 10.3389/fcvm.2023.1111475 (PMC9909180; doi:10.3389/fcvm.2023.1111475)

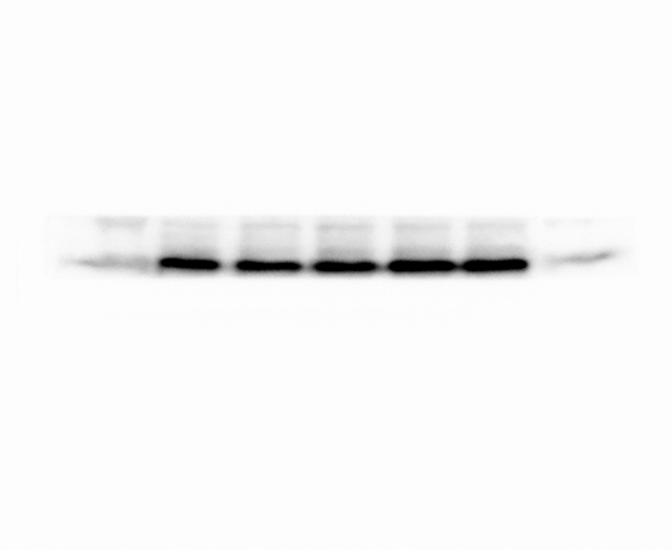

Supplement: Supplementary file 1 [file Data_Sheet_1.ZIP › western blot/Figure 10-G/GAPDH-1.tif]

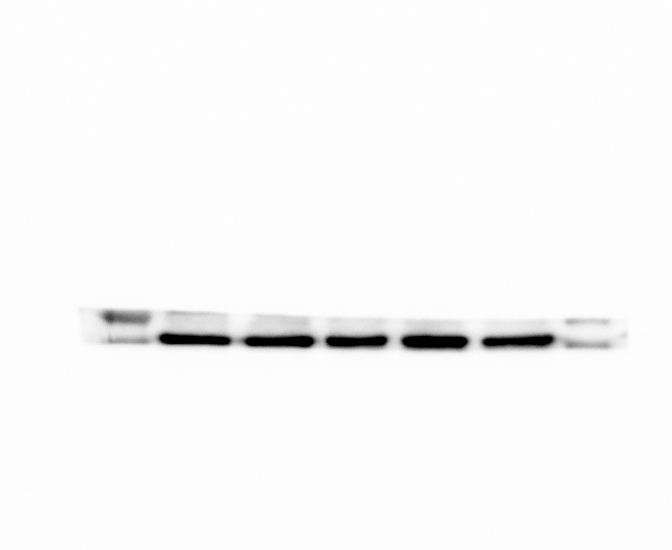

Supplement: Supplementary file 1 [file Data_Sheet_1.ZIP › western blot/Figure 10-G/GAPDH-2.tif]

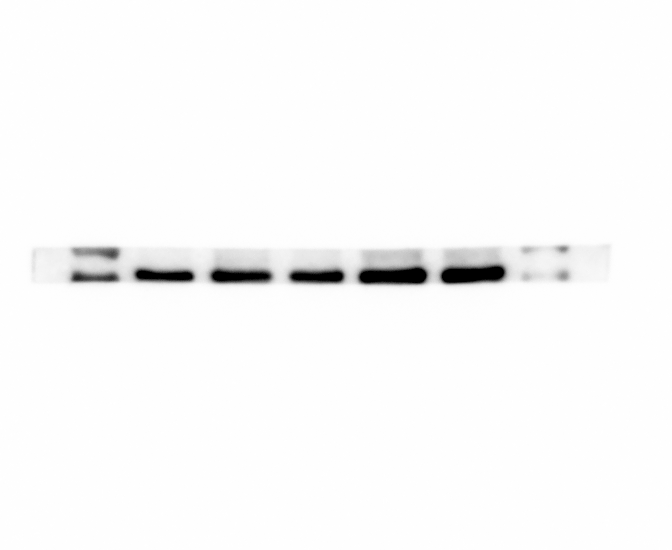

Supplement: Supplementary file 1 [file Data_Sheet_1.ZIP › western blot/Figure 10-G/GAPDH-3.tif]

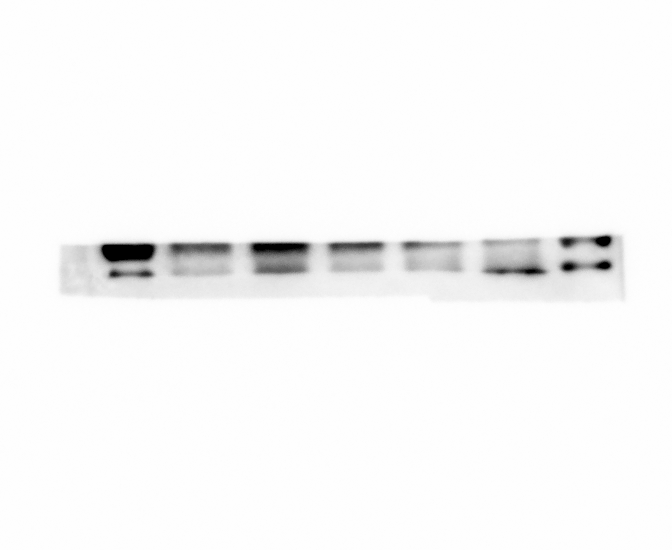

Supplement: Supplementary file 1 [file Data_Sheet_1.ZIP › western blot/Figure 10-G/IL18-1.tif]

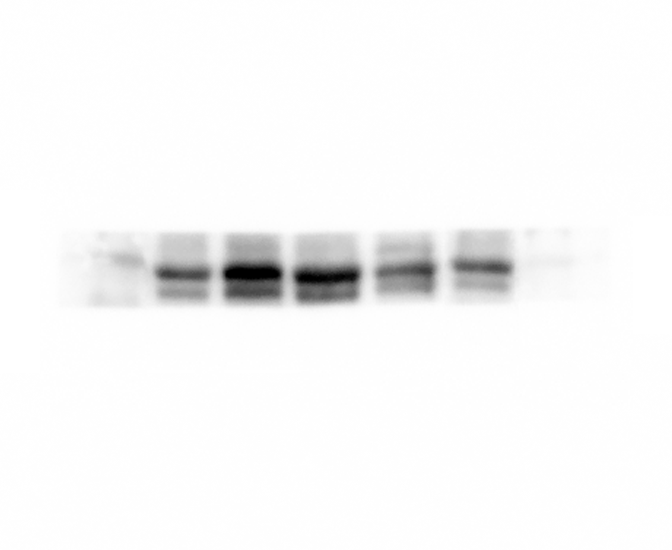

Supplement: Supplementary file 1 [file Data_Sheet_1.ZIP › western blot/Figure 10-G/IL18-2.tif]

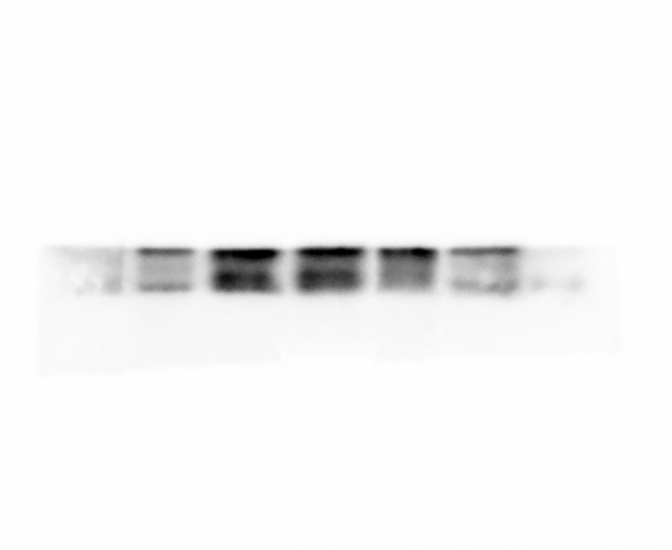

Supplement: Supplementary file 1 [file Data_Sheet_1.ZIP › western blot/Figure 10-G/IL18-3.tif]

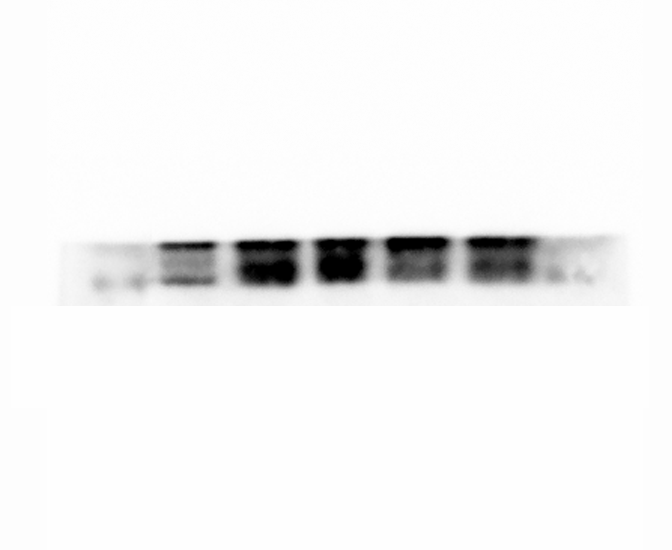

Supplement: Supplementary file 1 [file Data_Sheet_1.ZIP › western blot/Figure 10-G/IL1a┬-1.tif]

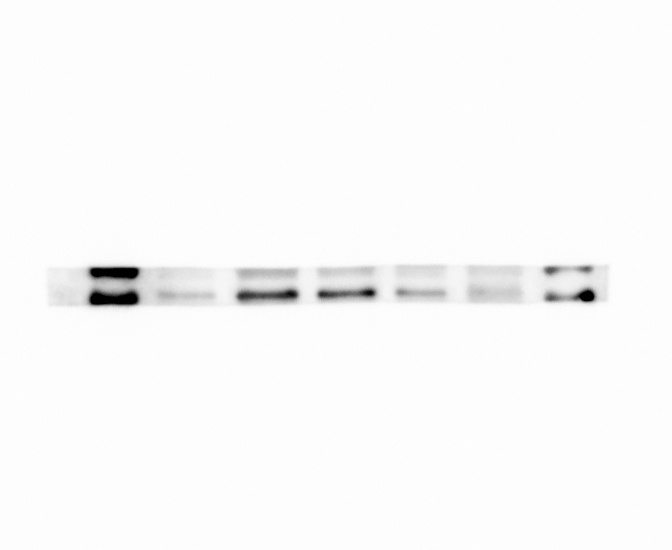

Supplement: Supplementary file 1 [file Data_Sheet_1.ZIP › western blot/Figure 10-G/IL1a┬-2.tif]

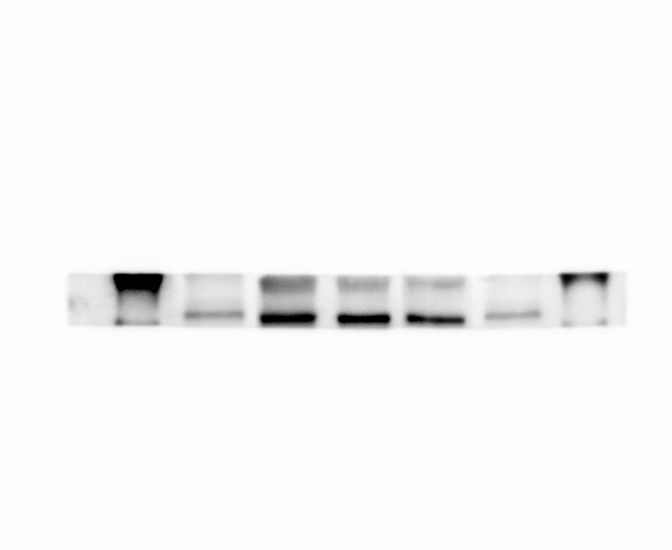

Supplement: Supplementary file 1 [file Data_Sheet_1.ZIP › western blot/Figure 10-G/IL1a┬-3.tif]

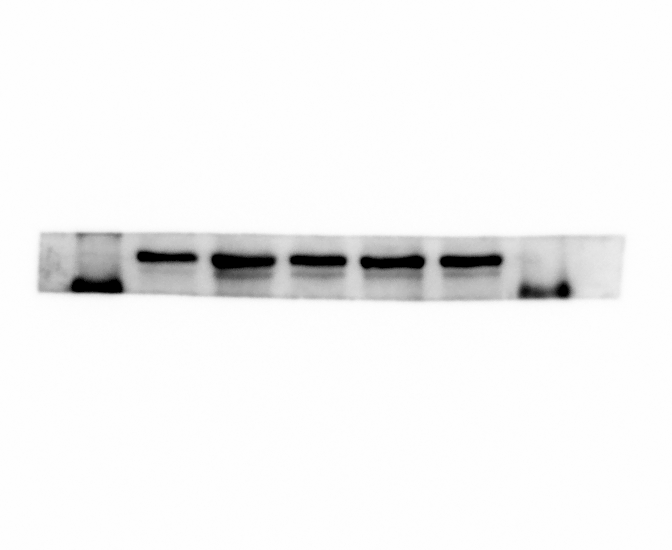

Supplement: Supplementary file 1 [file Data_Sheet_1.ZIP › western blot/Figure 10-G/NFa╩B-1.tif]

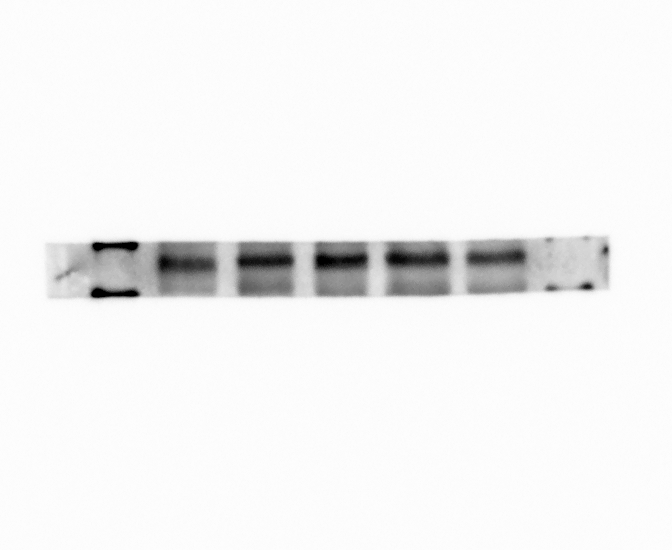

Supplement: Supplementary file 1 [file Data_Sheet_1.ZIP › western blot/Figure 10-G/NFa╩B-2.tif]

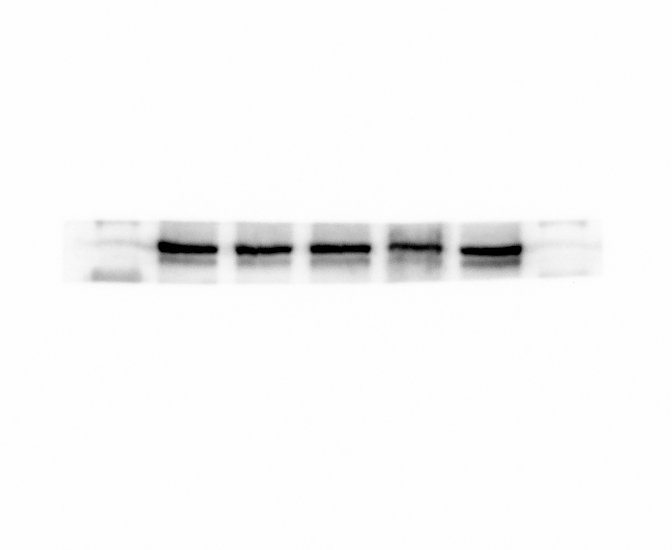

Supplement: Supplementary file 1 [file Data_Sheet_1.ZIP › western blot/Figure 10-G/NFa╩B-3.tif]

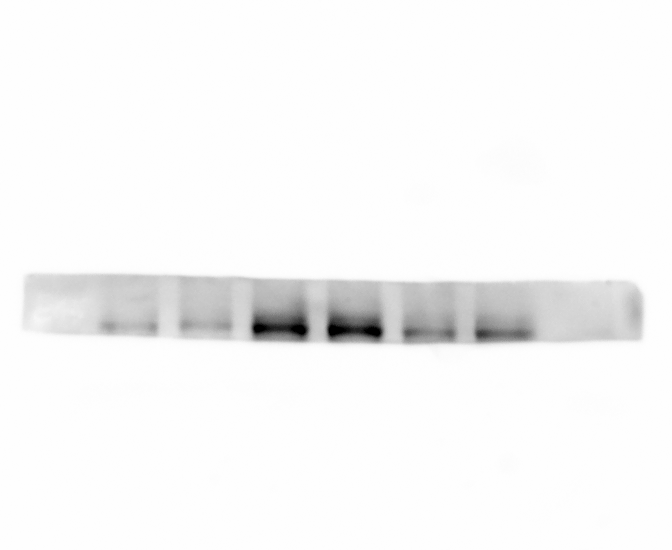

Supplement: Supplementary file 1 [file Data_Sheet_1.ZIP › western blot/Figure 10-G/TNFa-1.tif]

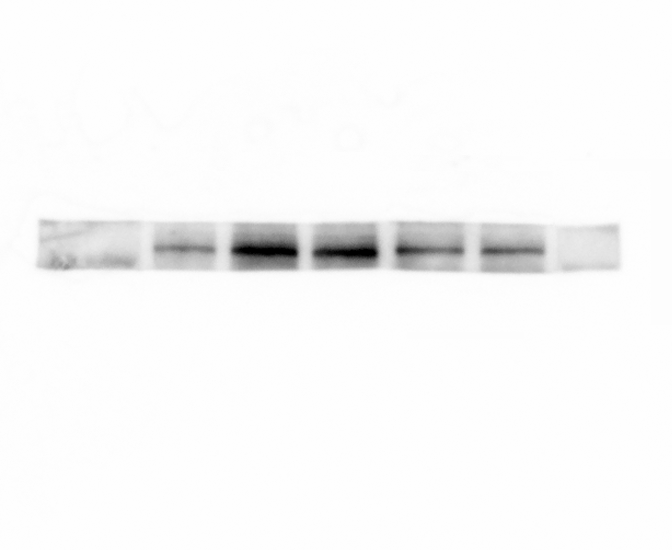

Supplement: Supplementary file 1 [file Data_Sheet_1.ZIP › western blot/Figure 10-G/TNFa-2.tif]

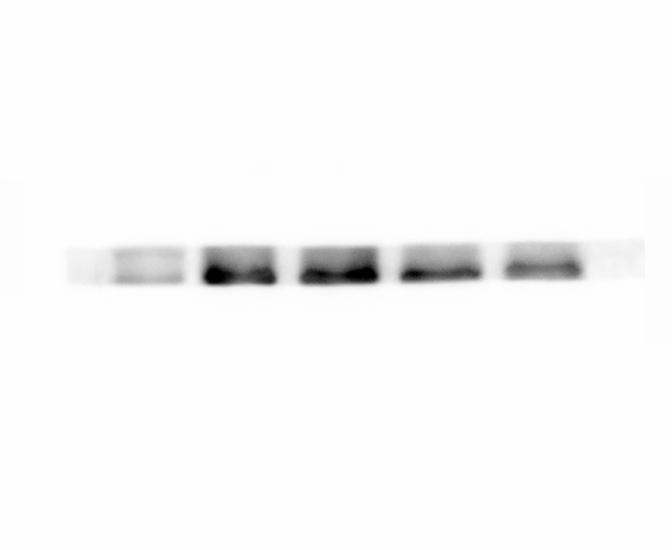

Supplement: Supplementary file 1 [file Data_Sheet_1.ZIP › western blot/Figure 10-G/TNFa-3.tif]

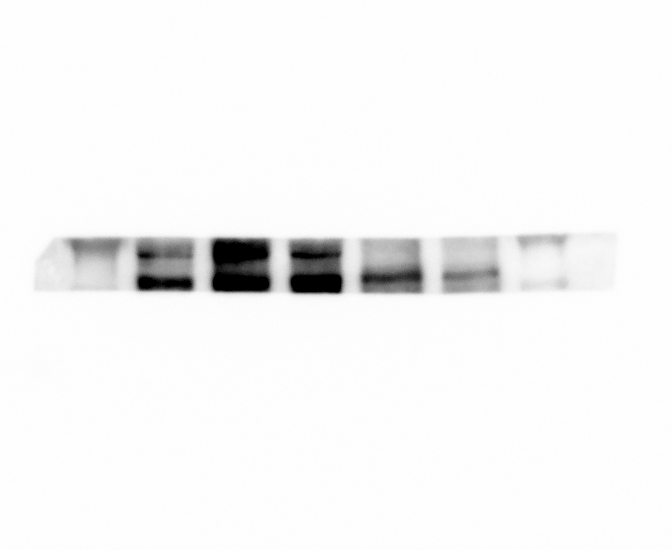

Supplement: Supplementary file 1 [file Data_Sheet_1.ZIP › western blot/Figure 10-G/pNFa╩B-1.tif]

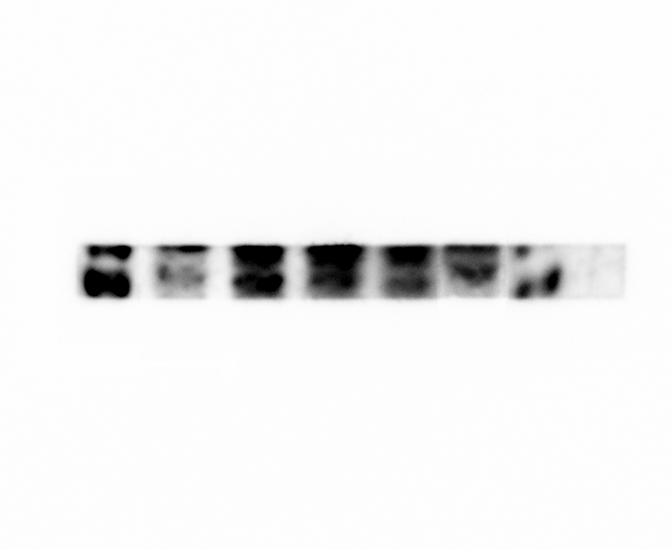

Supplement: Supplementary file 1 [file Data_Sheet_1.ZIP › western blot/Figure 10-G/pNFa╩B-2.tif]

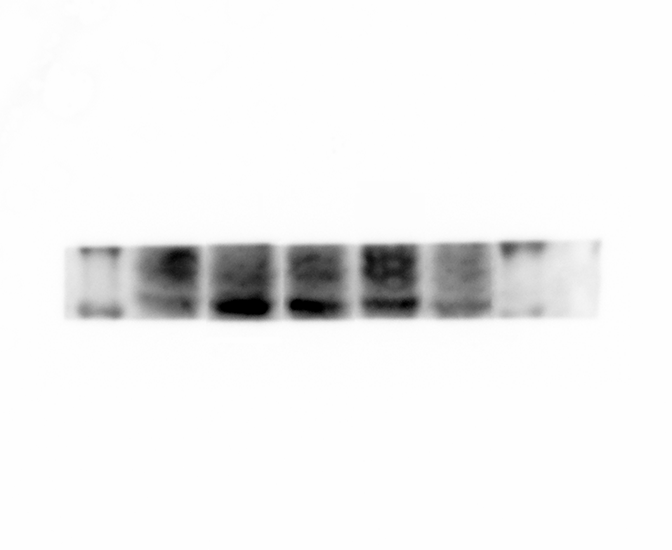

Supplement: Supplementary file 1 [file Data_Sheet_1.ZIP › western blot/Figure 10-G/pNFa╩B-3.tif]

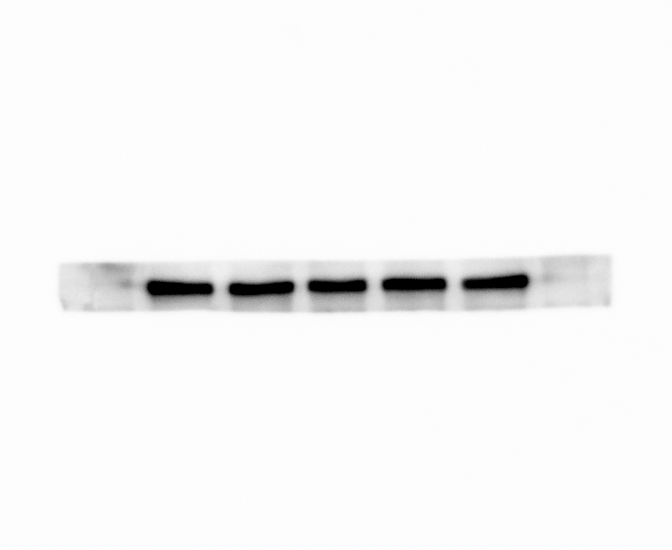

Supplement: Supplementary file 1 [file Data_Sheet_1.ZIP › western blot/Figure 11-H/AKT-1.tif]

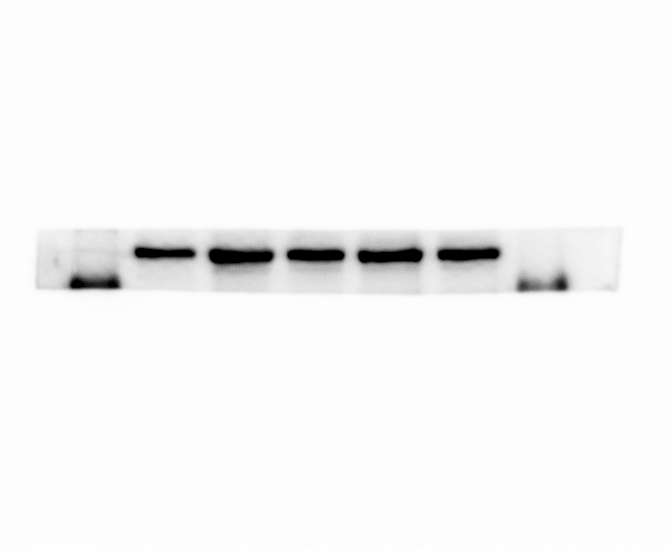

Supplement: Supplementary file 1 [file Data_Sheet_1.ZIP › western blot/Figure 11-H/AKT-2.tif]

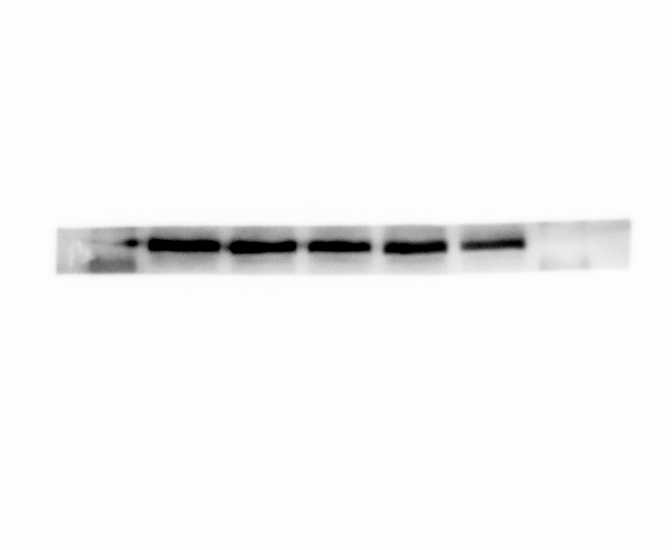

Supplement: Supplementary file 1 [file Data_Sheet_1.ZIP › western blot/Figure 11-H/AKT-3.tif]

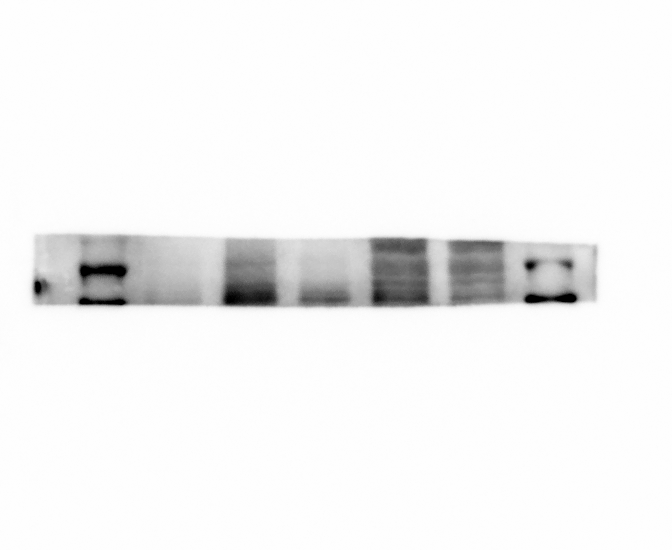

Supplement: Supplementary file 1 [file Data_Sheet_1.ZIP › western blot/Figure 11-H/FLT4-1.tif]

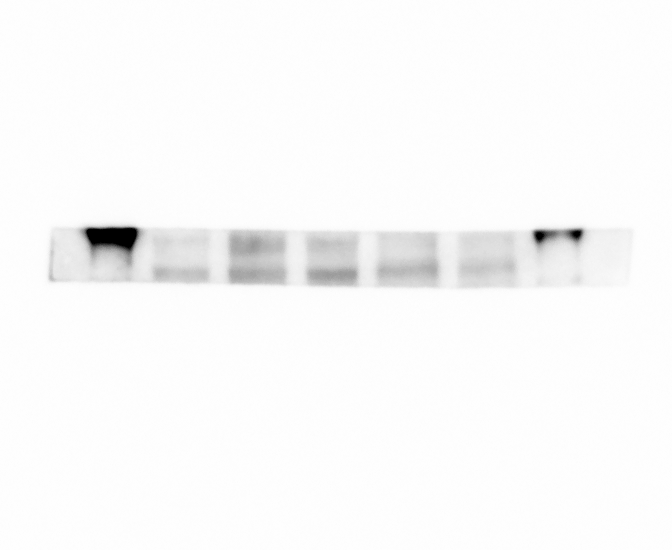

Supplement: Supplementary file 1 [file Data_Sheet_1.ZIP › western blot/Figure 11-H/FLT4-2.tif]

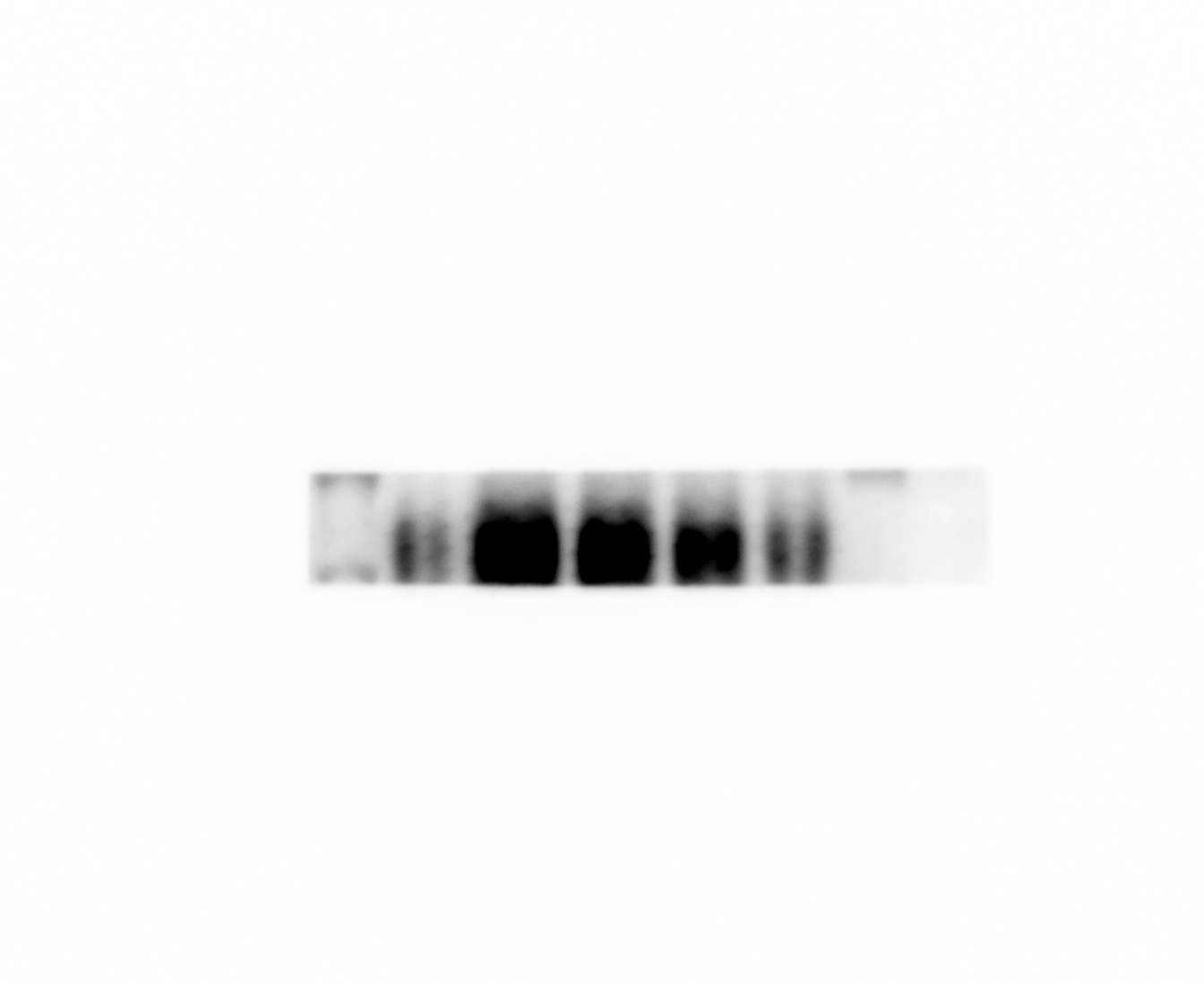

Supplement: Supplementary file 1 [file Data_Sheet_1.ZIP › western blot/Figure 11-H/FLT4-3.tif]

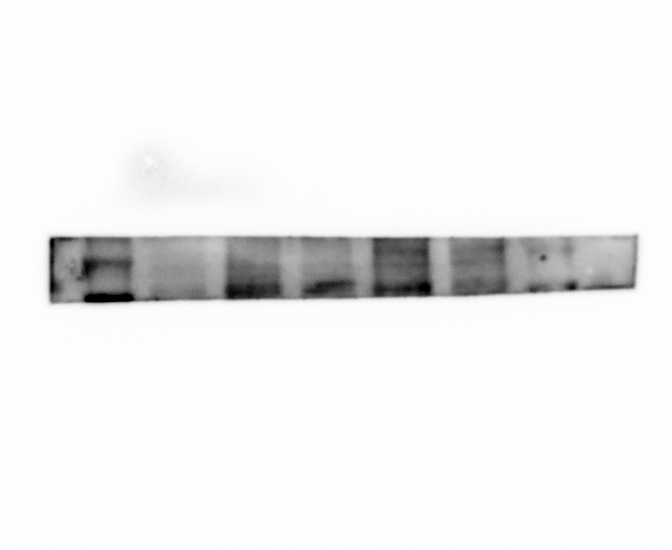

Supplement: Supplementary file 1 [file Data_Sheet_1.ZIP › western blot/Figure 11-H/HIF1a┴-1.tif]

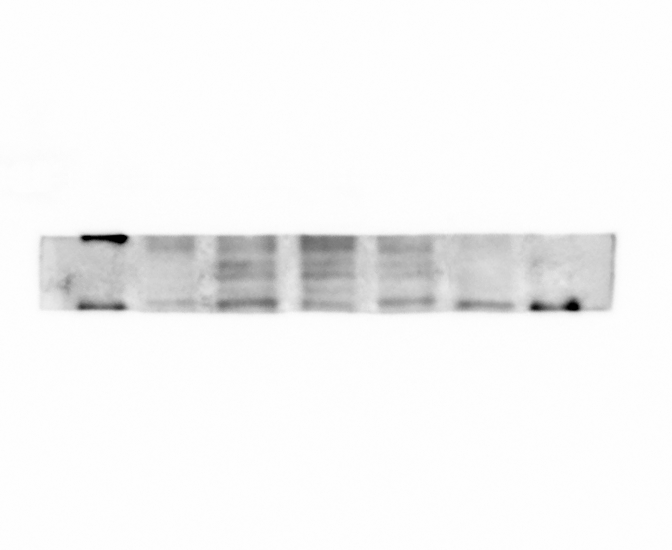

Supplement: Supplementary file 1 [file Data_Sheet_1.ZIP › western blot/Figure 11-H/HIF1a┴-2.tif]

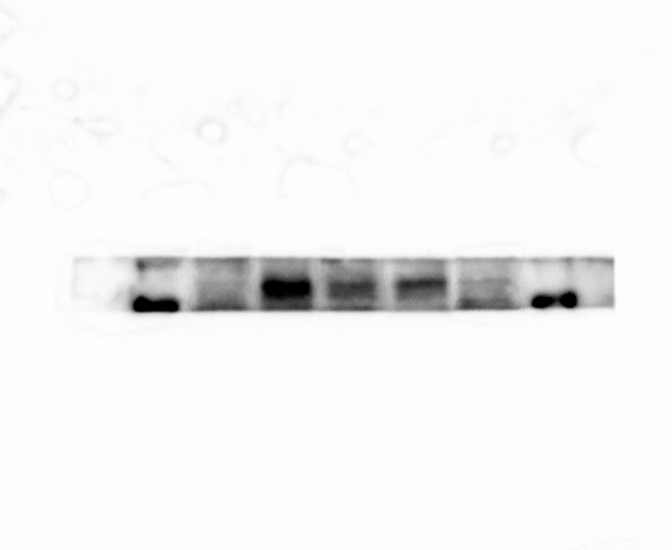

Supplement: Supplementary file 1 [file Data_Sheet_1.ZIP › western blot/Figure 11-H/HIF1a┴-3.tif]

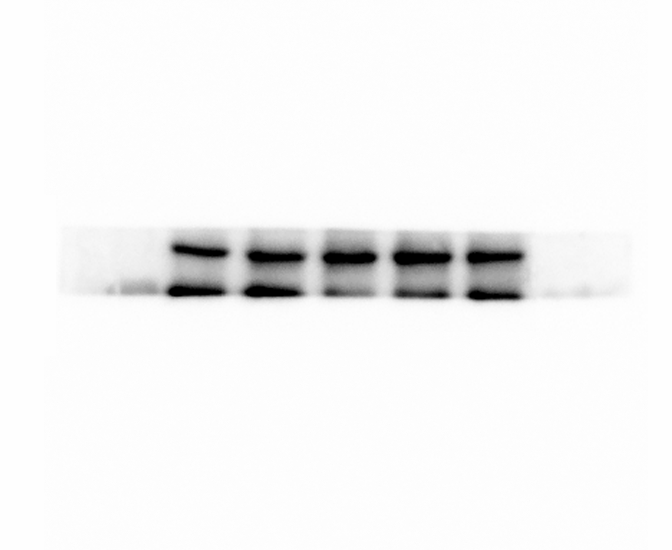

Supplement: Supplementary file 1 [file Data_Sheet_1.ZIP › western blot/Figure 11-H/P38-1.tif]

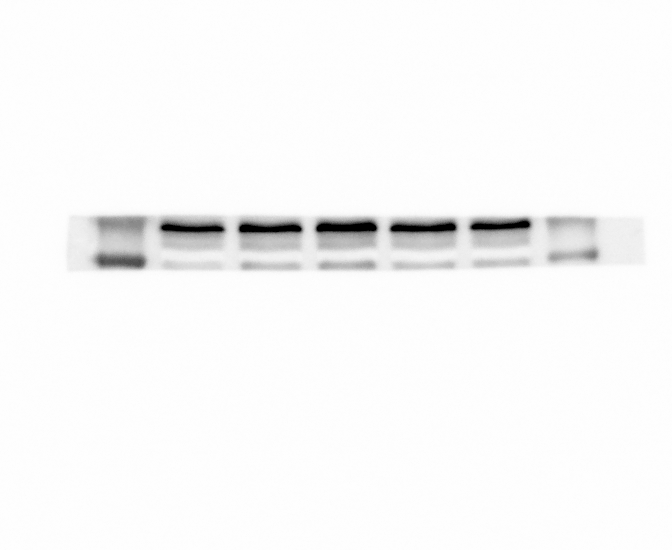

Supplement: Supplementary file 1 [file Data_Sheet_1.ZIP › western blot/Figure 11-H/P38-2.tif]

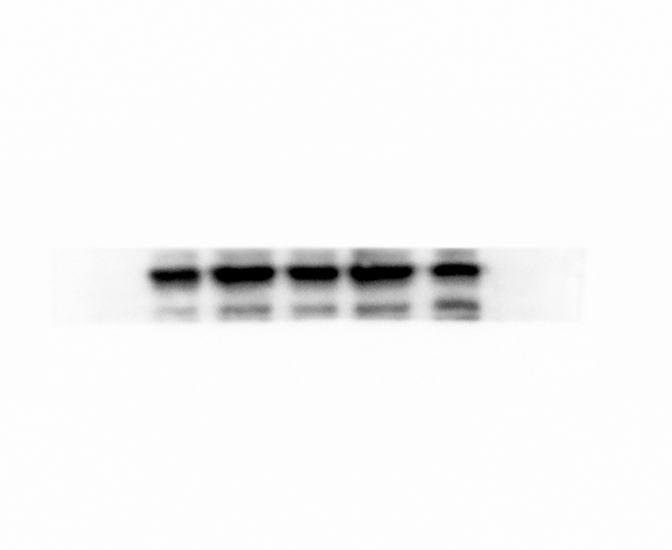

Supplement: Supplementary file 1 [file Data_Sheet_1.ZIP › western blot/Figure 11-H/P38-3.tif]

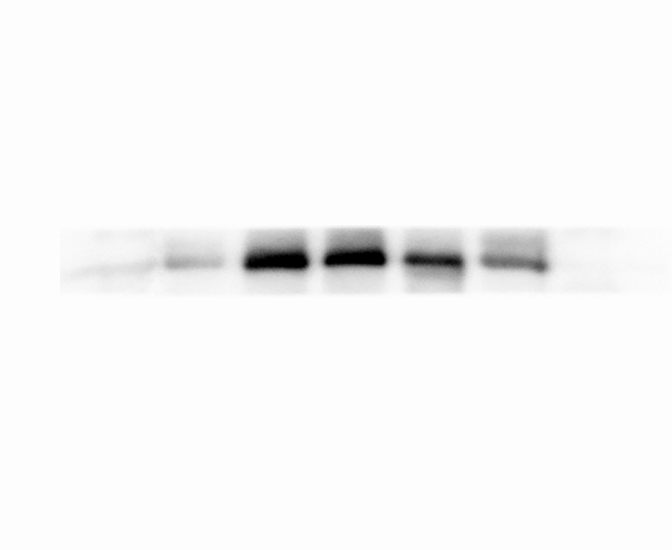

Supplement: Supplementary file 1 [file Data_Sheet_1.ZIP › western blot/Figure 11-H/PAKT-1.tif]

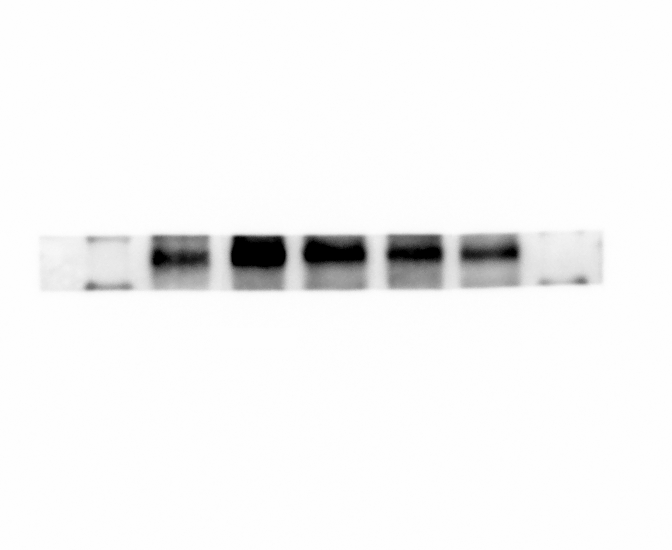

Supplement: Supplementary file 1 [file Data_Sheet_1.ZIP › western blot/Figure 11-H/PAKT-2.tif]

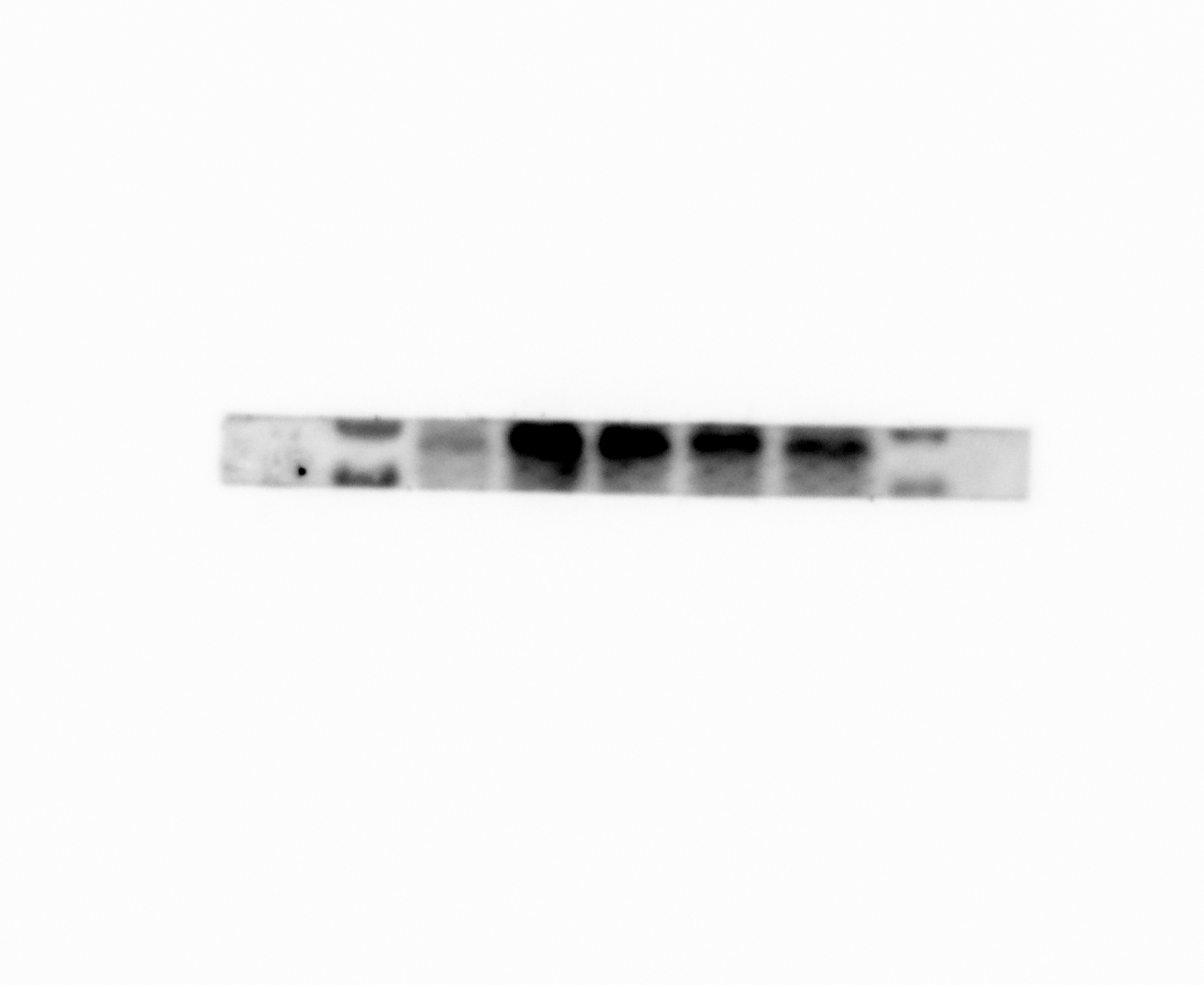

Supplement: Supplementary file 1 [file Data_Sheet_1.ZIP › western blot/Figure 11-H/PAKT-3.tif]

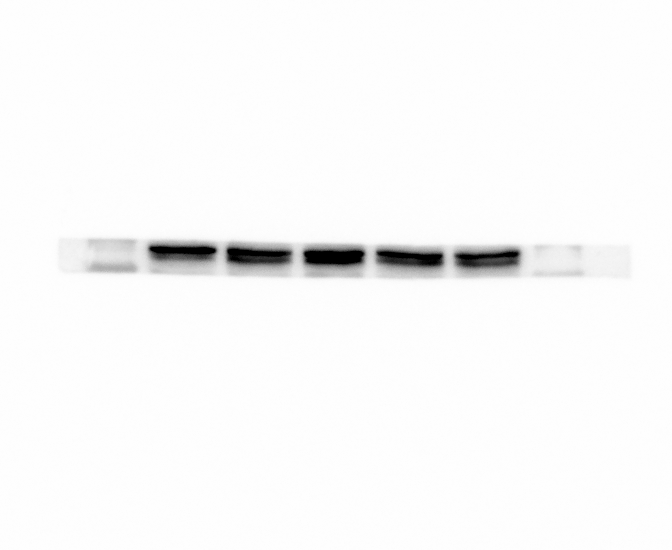

Supplement: Supplementary file 1 [file Data_Sheet_1.ZIP › western blot/Figure 11-H/PI3K-1.tif]

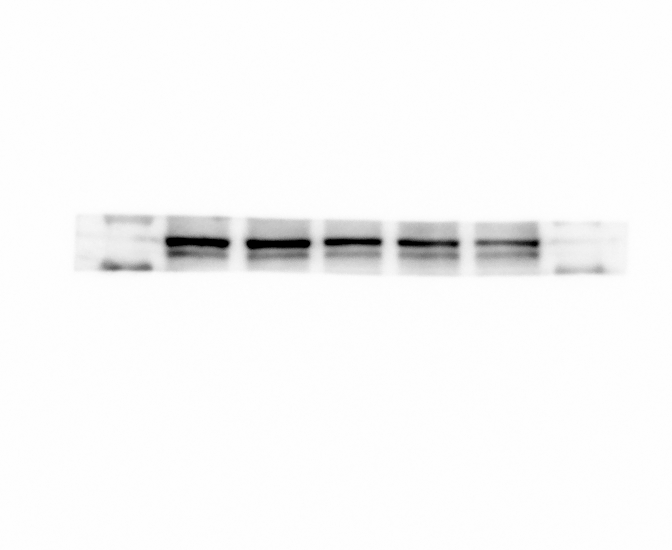

Supplement: Supplementary file 1 [file Data_Sheet_1.ZIP › western blot/Figure 11-H/PI3K-2.tif]

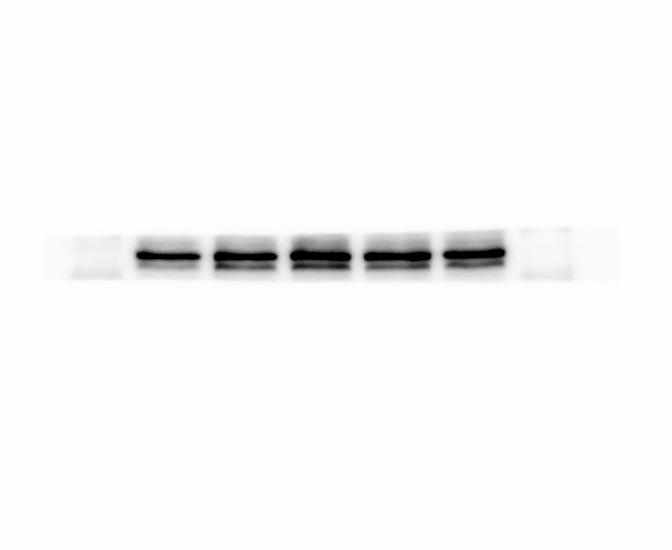

Supplement: Supplementary file 1 [file Data_Sheet_1.ZIP › western blot/Figure 11-H/PI3K-3.tif]

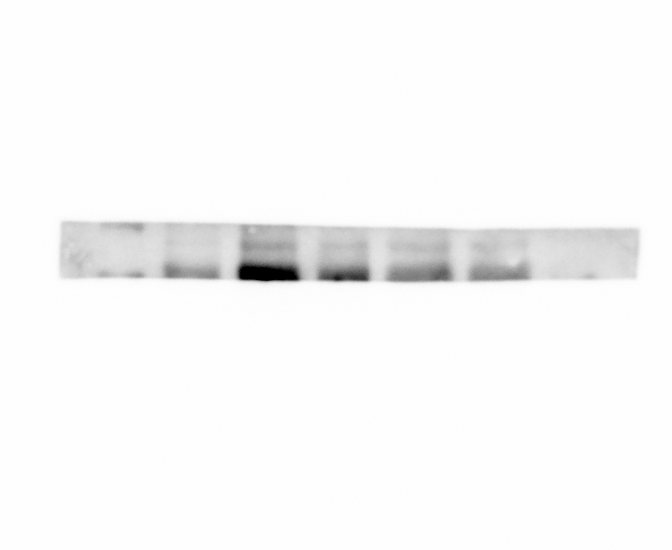

Supplement: Supplementary file 1 [file Data_Sheet_1.ZIP › western blot/Figure 11-H/PP38-1.tif]

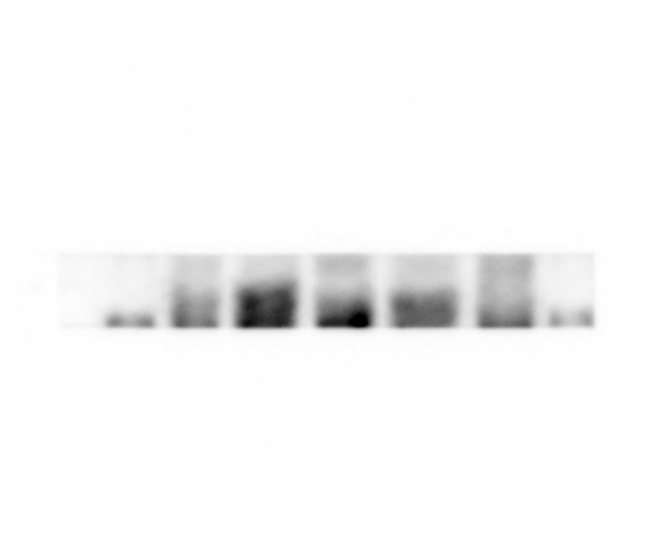

Supplement: Supplementary file 1 [file Data_Sheet_1.ZIP › western blot/Figure 11-H/PP38-2.tif]

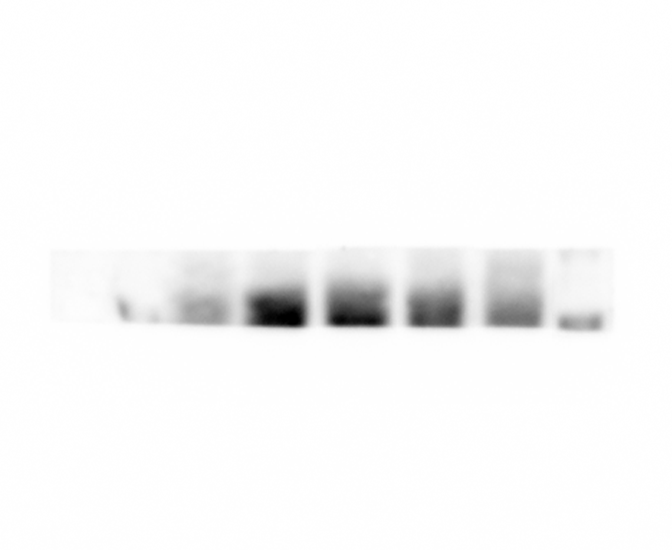

Supplement: Supplementary file 1 [file Data_Sheet_1.ZIP › western blot/Figure 11-H/PP38-3.tif]

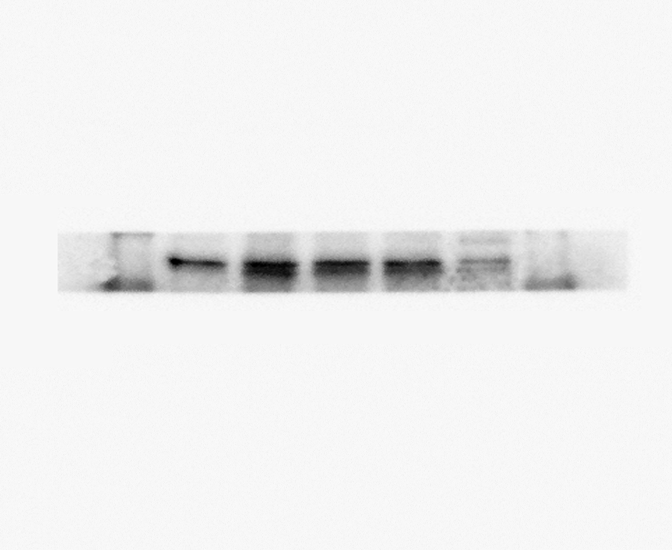

Supplement: Supplementary file 1 [file Data_Sheet_1.ZIP › western blot/Figure 11-H/PPI3K-1.tif]

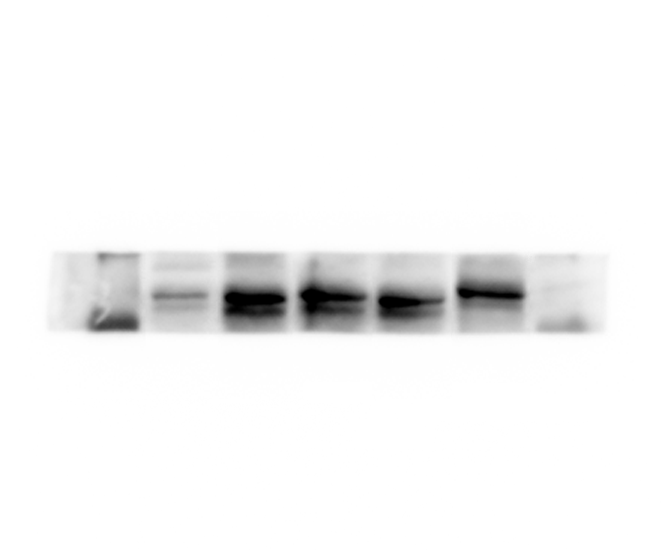

Supplement: Supplementary file 1 [file Data_Sheet_1.ZIP › western blot/Figure 11-H/PPI3K-2.tif]

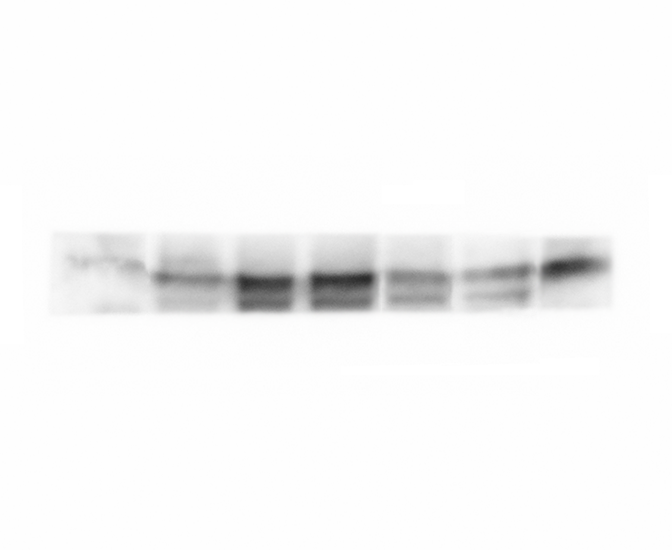

Supplement: Supplementary file 1 [file Data_Sheet_1.ZIP › western blot/Figure 11-H/PPI3K-3.tif]

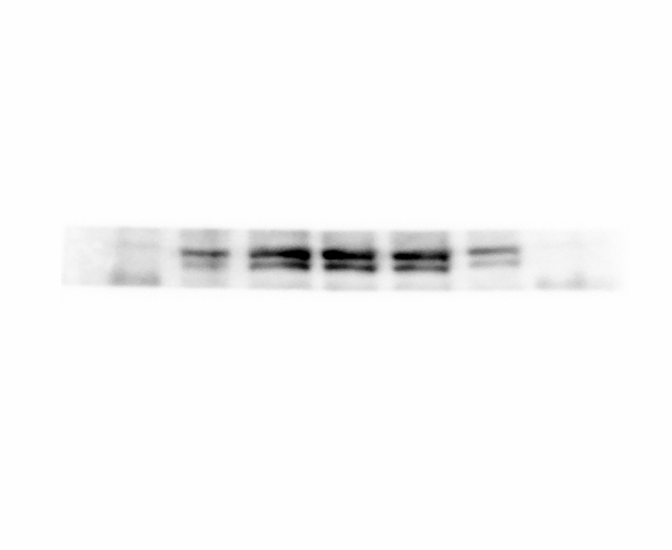

Supplement: Supplementary file 1 [file Data_Sheet_1.ZIP › western blot/Figure 11-H/PTGS2-1.tif]

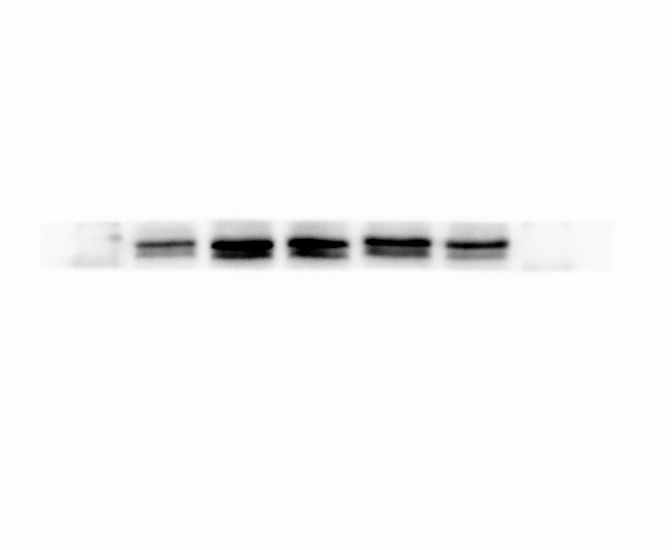

Supplement: Supplementary file 1 [file Data_Sheet_1.ZIP › western blot/Figure 11-H/PTGS2-2.tif]

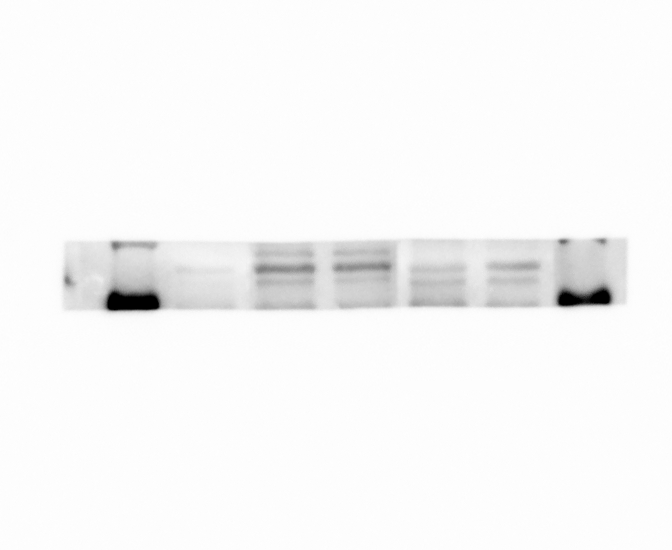

Supplement: Supplementary file 1 [file Data_Sheet_1.ZIP › western blot/Figure 11-H/PTGS2-3.tif]

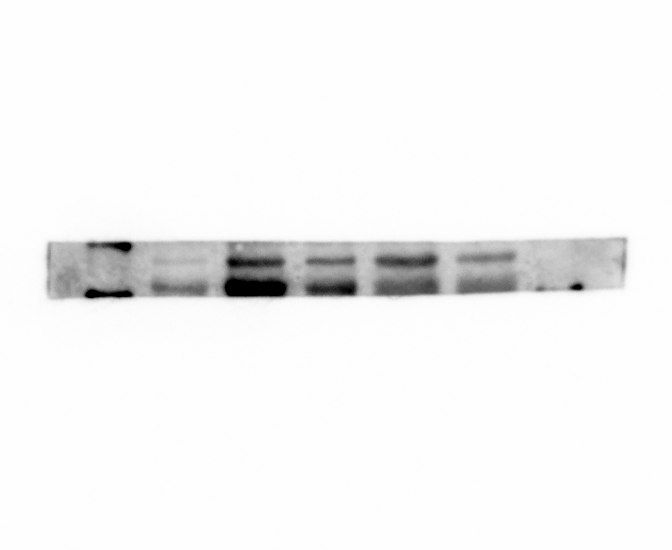

Supplement: Supplementary file 1 [file Data_Sheet_1.ZIP › western blot/Figure 11-H/VEGFA-1.tif]

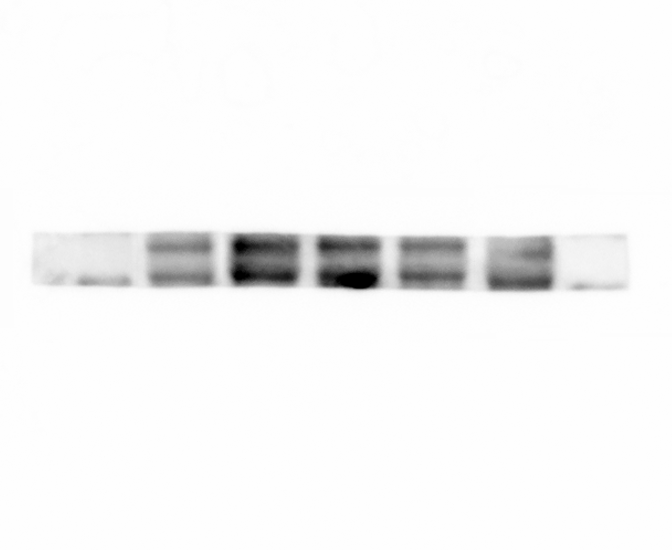

Supplement: Supplementary file 1 [file Data_Sheet_1.ZIP › western blot/Figure 11-H/VEGFA-2.tif]

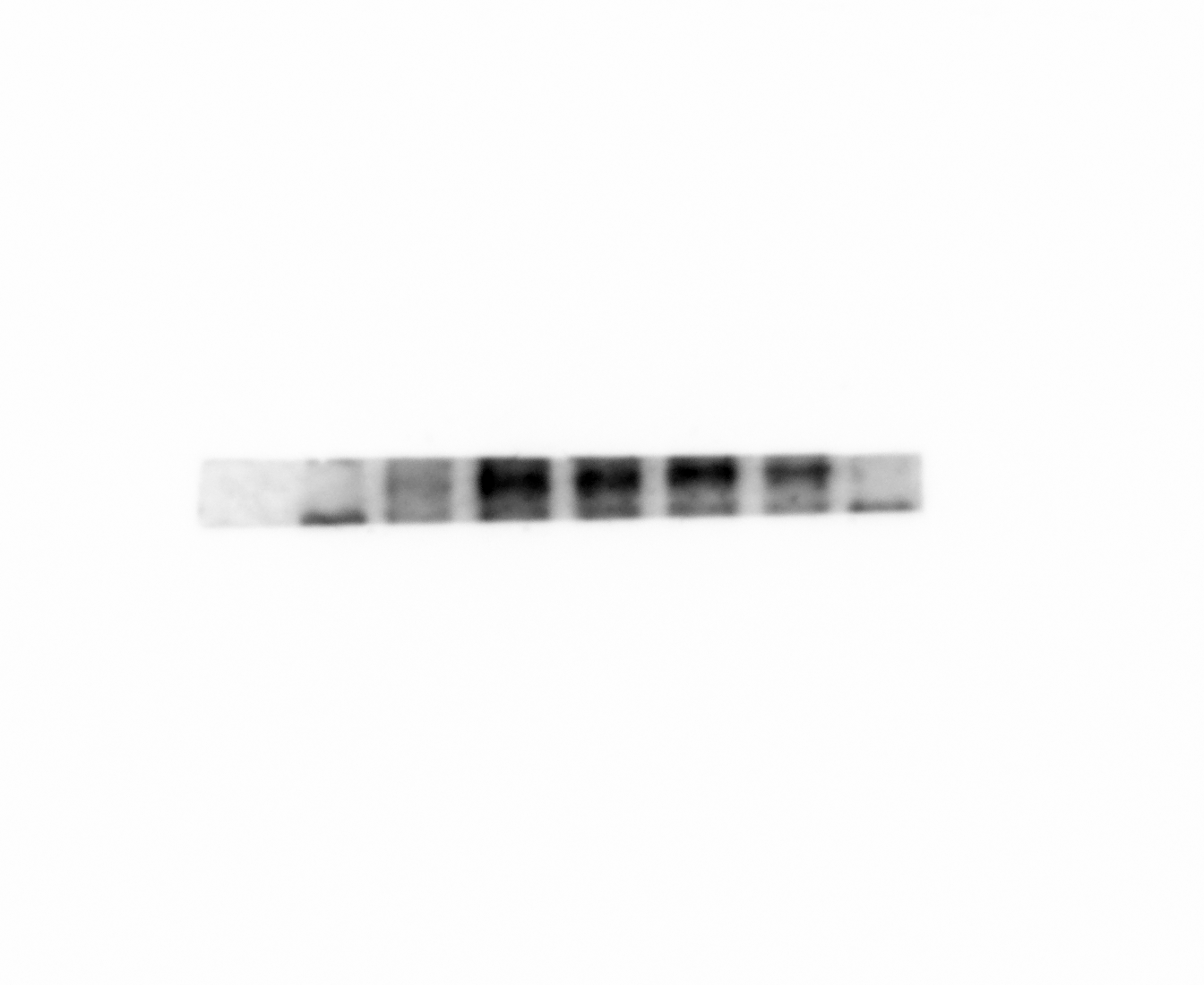

Supplement: Supplementary file 1 [file Data_Sheet_1.ZIP › western blot/Figure 11-H/VEGFA-3.tif]

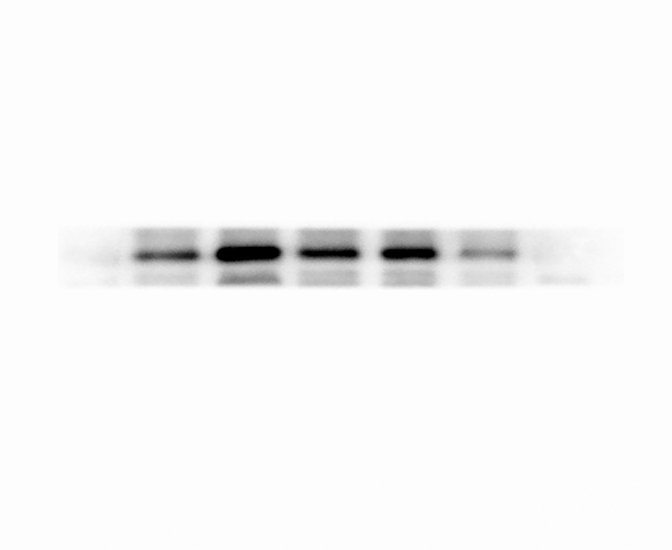

Supplement: Supplementary file 1 [file Data_Sheet_1.ZIP › western blot/Figure 11-H/VEGFC-1.tif]

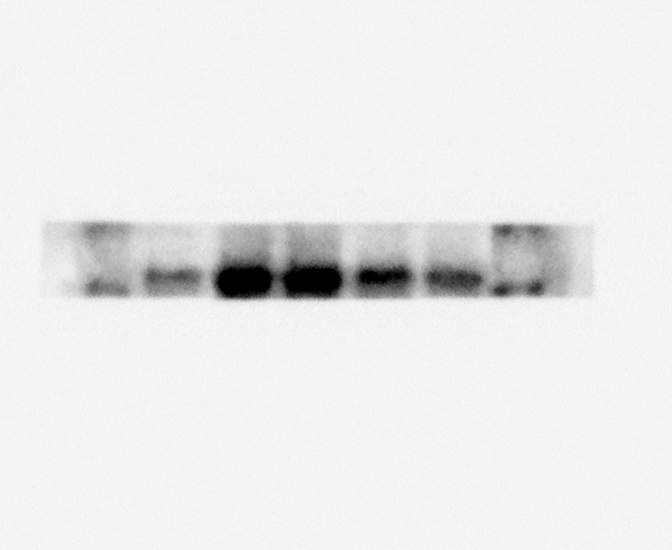

Supplement: Supplementary file 1 [file Data_Sheet_1.ZIP › western blot/Figure 11-H/VEGFC-2.tif]

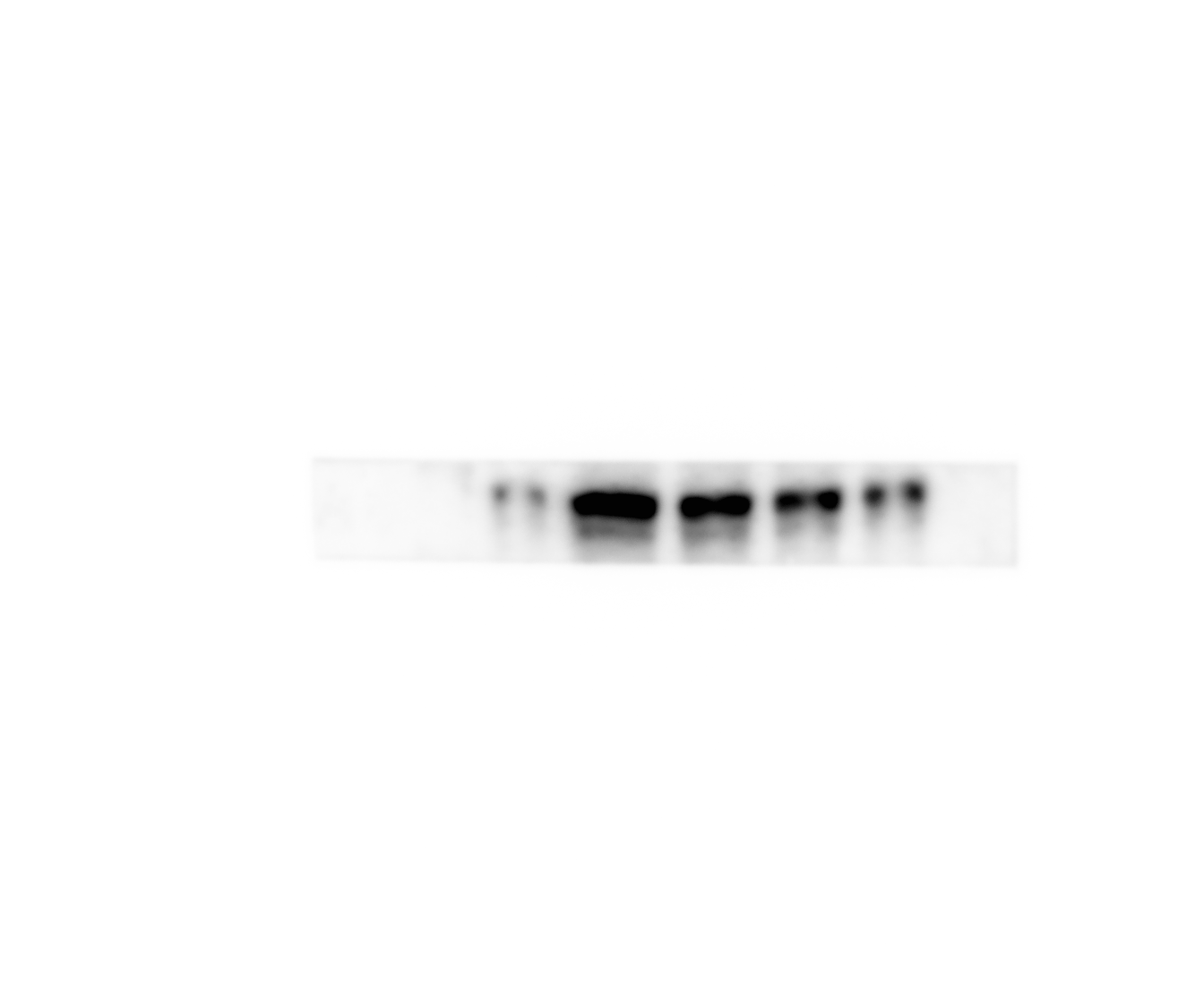

Supplement: Supplementary file 1 [file Data_Sheet_1.ZIP › western blot/Figure 11-H/VEGFC-3.tif]

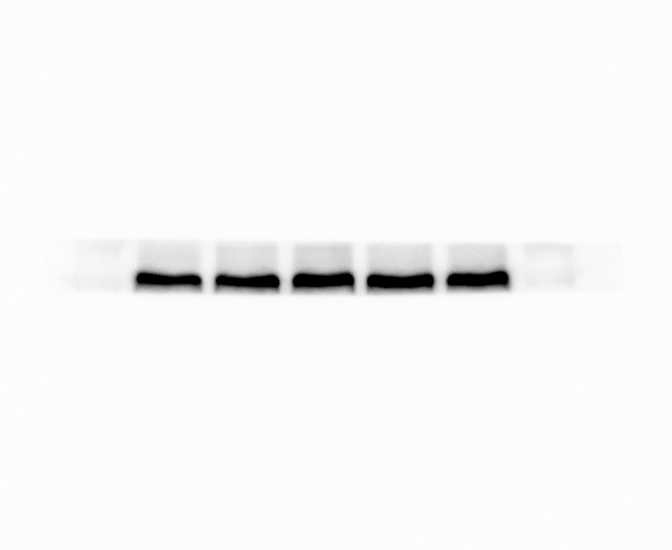

Supplement: Supplementary file 1 [file Data_Sheet_1.ZIP › western blot/Figure 11-H/a┴TUBLIN-1.tif]

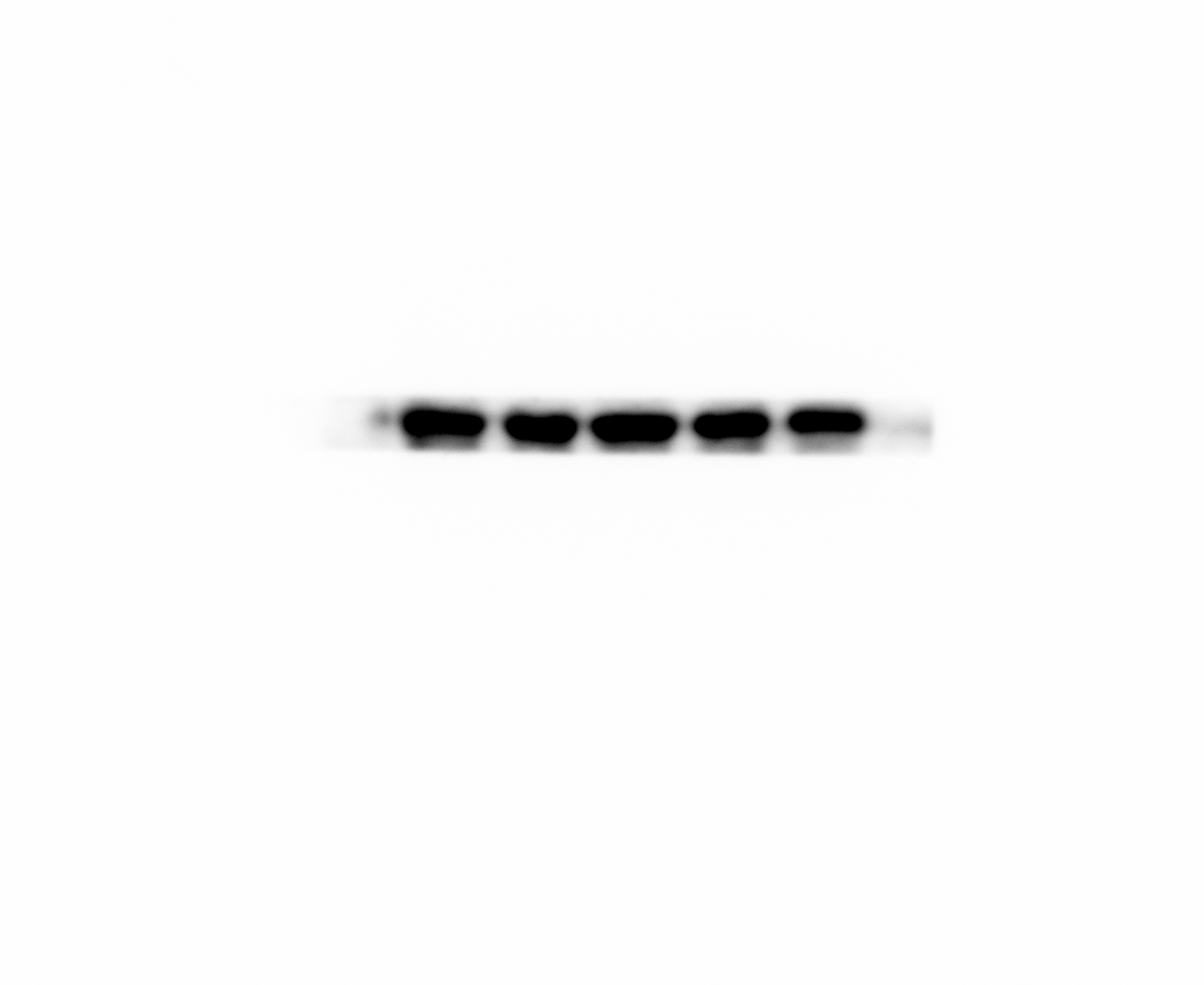

Supplement: Supplementary file 1 [file Data_Sheet_1.ZIP › western blot/Figure 11-H/a┴TUBLIN-2.tif]

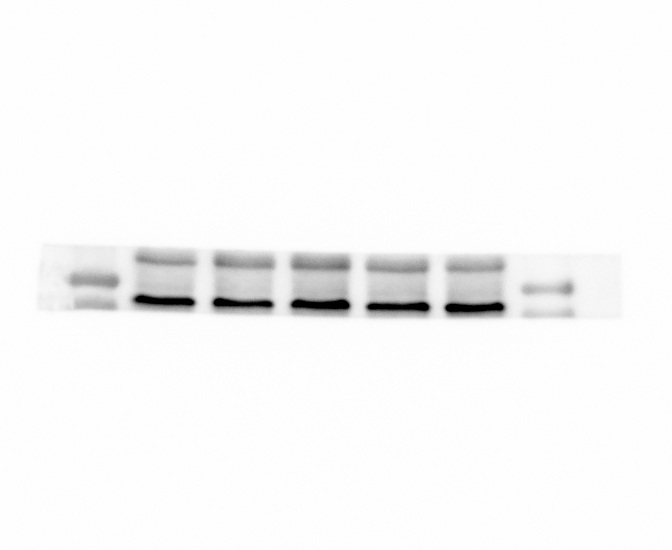

Supplement: Supplementary file 1 [file Data_Sheet_1.ZIP › western blot/Figure 11-H/a┴TUBLIN-3.tif]
