# Supplementary material for: Mechanism of Tao Hong Decoction in the treatment of atherosclerosis based on network pharmacology and experimental validation
Source: Front Cardiovasc Med. 2023 Jan 26;10:1111475. doi: 10.3389/fcvm.2023.1111475 (PMC9909180; doi:10.3389/fcvm.2023.1111475)
Supplement: Supplementary file 2 [file Table_1.DOCX]

**Mechanism of Tao Hong Decoction in the Treatment of Atherosclerosis Based on** **Network Pharmacology** **and** **Experimental Validation**

SiJin Li, Ping Liu*, Xiaoteng Feng, Min Du, Yifan Zhang, YiRu Wang, JiaRou Wang

*** Correspondence:**Ping Liu:liuping0207@yeah.net

# Supplementary Tables

| Table S1 Eluent gradient | | |
| --- | --- | --- |
| Time (min) | A% | B% |
| 0～3 | 0 | 100 |
| 3～7 | 0～5 | 100～95 |
| 7～10 | 5～10 | 95～90 |
| 10～13 | 10 | 90 |
| 13～30 | 10～40 | 90～60 |
| 30～33 | 40～95 | 60～5 |
| 33～36 | 95 | 5 |
| 36～36.1 | 95～0 | 5～100 |
| 36.1～40 | 0 | 100 |

| Table S2 Mass parameters (Sciex Triple TOF 4600 LC-MS) | | | |
| --- | --- | --- | --- |
| MS parameter | parameter values | MS/MS parameter | parameter values |
| TOF mass range | 50～1700 | MS/MS mass range | 50～1250 |
| Ion Source Gas 1（psi） | 50 | Declustering Potential（V） | 100 |
| Ion Source Gas 2（psi） | 50 | Collision Energy（eV） | ±40 |
| Curtain Gas（psi） | 35 | Collision Energy Spread（eV） | 20 |
| Ion Spray Voltage Floating (V) | -4500/5000 | Ion Release Delay（ms） | 30 |
| Ion Source Temperature (°C) | 500 | Ion Release Width（ms） | 15 |
| Declustering Potential（V） | 100 |  |  |
| Collision Energy（eV） | 10 |  |  |

| Table S3 Sequences of primers used for RT-qPCR analysis. | | |
| --- | --- | --- |
| Primer Name | Forward Sequence (5′→ 3′) | Reverse Sequence (5′→ 3′) |
| IL-6 | CCAAGAGGTGAGTGCTTCCC | CTGTTGTTCAGACTCTCTCCCT |
| IL-1α | GCACCTTACACCTACCAGAGT | AAACTTCTGCCTGACGAGCTT |
| TNF-α | CCCTCACACTCAGATCATCTTCT | GCTACGACGTGGGCTACAG |
| PTGS2 | TGAGCAACTATTCCAAACCAGC | GCACGTAGTCTTCGATCACTATC |
| HIF-1α | ACCTTCATCGGAAACTCCAAAG | ACTGTTAGGCTCAGGTGAACT |
| VEGFC | GAGGTCAAGGCTTTTGAAGGC | CTGTCCTGGTATTGAGGGTGG |
| VEGFA | GCACATAGAGAGAATGAGCTTCC | CTCCGCTCTGAACAAGGCT |
| FLT-4 | CTGGCAAATGGTTACTCCATGA | ACAACCCGTGTGTCTTCACTG |
| AKT1 | ATGAACGACGTAGCCATTGTG | TTGTAGCCAATAAAGGTGCCAT |
| CTNNB1 | ATGGAGCCGGACAGAAAAGC | TGGGAGGTGTCAACATCTTCTT |
| NF-kB | GGAGGCATGTTCGGTAGTGG | CCCTGCGTTGGATTTCGTG |

| Table S4 Results of identification of main components of Taohong drink samples | | | | | | | | | | |
| --- | --- | --- | --- | --- | --- | --- | --- | --- | --- | --- |
| Serial number | Time （min） | Adduct-ion | *m/z* actual value | *m/z*  theoretical value | ppm | molecular formula | molecular weight | English name | MS/MS data | Affiliation |
| 1 | 2.30 | [M-H]^-^ | 499.167 | 499.1668 | 0.3 | C_19_H_32_O_15_ | 500.17 | 3-{[6-O-(D-galactopyranosyl)-β-D-galactopyranosyl] oxy}-1, 2-propanediyl diacetate | 191.0567;173.0459;93.0356 | Honghua |
| 2 | 5.62 | [M-H]^-^ | 282.0837 | 282.0844 | -2.5 | C_10_H_13_N_5_O_5_ | 283.09 | Guanosine | 282.0847;150.0423;133.0158;108.0208 | All |
| 3 | 9.41 | [M+H]^+^ | 476.1762 | 476.1763 | -0.1 | C_20_H_29_NO_12_ | 475.17 | D-(-)-mandelamideO-β-D-Gentiobioside | 152.0693;134.0592;106.0642 | Taoren |
| 4 | 9.58 | [M-H]^-^ | 611.1614 | 611.1618 | -0.1 | C_27_H_32_O_16_ | 612.17 | Hydroxysafflor yellow B | 611.1635;491.1214;473.1093;303.0722;283.0624 | Honghua |
| 5 | 10.85 | [M-H]^-^ | 475.1451 | 475.1457 | -1.3 | C_20_H_28_O_13_ | 476.15 | Amygdalinic acid | 475.1451;431.1552;269.1018;161.0451 | Taoren |
| 6 | 11.94 | [M+FA-H]^-^ | 477.1614 | 477.1614 | 0.1 | C_19_H_28_O_11_ | 432.16 | Benzyl Gentiobioside | 431.1550;269.1025;161.0460 | Taoren |
| 7 | 12.17 | [M-H]^-^ | 353.0872 | 353.0878 | -1.7 | C_16_H_18_O_9_ | 354.10 | Chlorogenic acid | 191.0570;179.0353;173.0470;135.0453 | Danggui, Chuanxiong , Taoren, Honghua |
| 8 | 12.38 | [M-H]^-^ | 801.1725 | 801.1731 | -0.8 | C_33_H_38_O_23_ | 802.18 | 6-hydroxykaempferol 3,6-di-O-β-d-glucoside-7-O-β-d-glucuronide | 801.1703;625.1405;463.0887;301.0360 | Honghua |
| 9 | 12.47 | [M+FA-H]^-^ | 502.1567 | 502.1566 | 0.2 | C_20_H_27_NO_11_ | 457.16 | L-Amygdalin | 456.1535;323.0974;221.0662;179.0557;161.0456 | Taoren |
| 10 | 12.50 | [M-H]^-^ | 611.162 | 611.1618 | 0.4 | C_27_H_32_O_16_ | 612.17 | Hydroxysafflor yellow A | 611.1601;491.1191;403.1031;325.0712;283.0606 | Honghua |
| 11 | 12.60 | [M+FA-H]^-^ | 502.1557 | 502.1566 | -1.8 | C_20_H_27_NO_11_ | 457.16 | D-Amygdalin | 456.1476;323.0984;221.0683;179.0569;161.0460 | Taoren |
| 12 | 15.91 | M^+^ | 342.1701 | 342.17 | 0.4 | C_20_H_24_NO_4_^+^ | 342.17 | Magnoflorine | 297.1116;282.0882;265.0850;222.0670;191.0855 | Weilingxian |
| 13 | 17.52 | [M+H]^+^ | 195.0656 | 195.0652 | 2.1 | C_10_H_10_O_4_ | 194.06 | Ferulic acid | 177.0540;149.0589;145.0247;134.0355;117.0328 | Weilingxian ,Danggui , Chuanxiong |
| 14 | 17.62 | [M-H]^-^ | 625.1429 | 625.141 | 3 | C_27_H_30_O_17_ | 626.15 | 6-Hydroxykaempferol 3,6-Diglucoside | 625.1424;463.0889;301.0352;299.0190;271.0244 | Honghua |
| 15 | 18.47 | [M+H]^+^ | 195.0656 | 193.0552 | 2.1 | C_10_H_10_O_4_ | 194.06 | Isoferulic acid | 177.0540;149.0589;145.0274;134.0355;117.0328 | Weilingxian , Danggui ,Chuanxiong |
| 16 | 20.61 | [M-H]^-^ | 593.1515 | 593.1512 | 0.2 | C_27_H_30_O_15_ | 594.16 | Kaempferol-3-O-rutinoside | 593.1444;285.0373;284.0295;255.0275 | Taoren , Honghua |
| 17 | 20.88 | [M-H]^-^ | 623.1619 | 623.1618 | 0.2 | C_28_H_32_O_16_ | 624.17 | Narcissoside | 315.0492;314.0411;300.0264;299.0192;271.0226 | Taoren , Honghua |
| 18 | 22.10 | [M+H]^+^ | 679.5128 | 679.5143 | -2.3 | C_40_H_70_O_8_ | 678.51 | Cyclohexaleucyl(iso)leucyl | 661.5025; 435.3346;336.2283;209.1652 | All |
| 19 | 22.56 | [M-H]^-^ | 613.1564 | 613.1563 | 0.1 | C_30_H_30_O_14_ | 614.16 | Safflomin C | 407.0965;361.1065;287.0560;241.0503;207.0501; 119.0597 | Honghua |
| 20 | 27.46 | [M-H]^-^ | 1335.6607 | 1335.6591 | 1.2 | C_64_H_104_O_29_ | 1336.67 | Huzhangoside B | / | Weilingxian |
| 21 | 27.50 | [M-H_2_O+H]^+^ | 439.3575 | 439.3571 | 1.0 | C_30_H_48_O_3_ | 456.36 | Oleanolic acid | 439.3581;203.1796;191.1793;163.1491;133.0106 | Weilingxian |
| 22 | 33.22 | [M+H]^+^ | 191.1065 | 191.1067 | -0.8 | C_12_H_14_O_2_ | 190.10 | Ligustilide | 191.1066;173.0960;129.0597;115.0539;105.0596 | Danggui , Chuanxiong |

| Table S5 The core active ingredients of THD for the treatment of AS. | | | | | |
| --- | --- | --- | --- | --- | --- |
| Herb name | Molecule ID | Molecule name | OB(%) | DL | Degree |
| Flos Carthami  (Honghua) | MOL002714 | baicalein | 33.52 | 0.21 | 21 |
| Flos Carthami  (Honghua) | MOL002773 | beta-carotene | 37.18 | 0.58 | 15 |
| Rhizoma Ligustici  (Chuanxiong) | MOL002135 | Myricanone | 40.6 | 0.51 | 15 |
| Radix Angelicae Sinensis  (Danggui) | MOL000449 | Stigmasterol | 43.83 | 0.76 | 14 |
| Radix Clematidis  (Weilingxian) | MOL000358 | beta-sitosterol | 36.91 | 0.75 | 9 |
| Semen Persicae  (Taoren) | MOL000296 | hederagenin | 36.91 | 0.75 | 8 |
| Flos Carthami  (Honghua) | MOL002712 | 6-Hydroxykaempferol | 62.13 | 0.27 | 6 |
| Flos Carthami  (Honghua) | MOL002721 | quercetagetin | 45.01 | 0.31 | 4 |
| Rhizoma Ligustici  (Chuanxiong) | MOL002157 | wallichilide | 42.31 | 0.71 | 4 |
| Flos Carthami  (Honghua) | MOL002695 | lignan | 43.32 | 0.65 | 3 |
| Rhizoma Ligustici  (Chuanxiong) | MOL001494 | Mandenol | 42 | 0.19 | 3 |
| Semen Persicae  (Taoren) | MOL001368 | 3-O-p-coumaroylquinic acid | 37.63 | 0.29 | 3 |
| Semen Persicae  (Taoren) | MOL001323 | Sitosterol alpha1 | 43.28 | 0.78 | 3 |
| Semen Persicae  (Taoren) | MOL000493 | campesterol | 37.58 | 0.71 | 3 |
| Flos Carthami  (Honghua) | MOL002757 | 7,8-dimethyl-1H-pyrimido[5,6-g]quinoxaline-2,4-dione | 45.75 | 0.19 | 2 |
| Flos Carthami  (Honghua) | MOL002717 | qt_carthamone | 51.03 | 0.2 | 2 |
| Flos Carthami  (Honghua) | MOL002710 | Pyrethrin II | 48.36 | 0.35 | 2 |
| Flos Carthami  (Honghua) | MOL002694 | 4-[(E)-4-(3,5-dimethoxy-4-oxo-1-cyclohexa-2,5-dienylidene)but-2-enylidene]-2,6-dimethoxycyclohexa-2,5-dien-1-one | 48.47 | 0.36 | 2 |
| Rhizoma Ligustici  (Chuanxiong) | MOL002140 | Perlolyrine | 65.95 | 0.27 | 2 |
| Rhizoma Ligustici  (Chuanxiong) | MOL000359 | sitosterol | 36.91 | 0.75 | 2 |
| Semen Persicae  (Taoren) | MOL001358 | gibberellin 7 | 73.8 | 0.5 | 2 |
| Semen Persicae  (Taoren) | MOL001352 | GA54 | 64.21 | 0.53 | 2 |
| Semen Persicae  (Taoren) | MOL001329 | 2,3-didehydro GA77 | 88.08 | 0.53 | 2 |
| Semen Persicae  (Taoren) | MOL001328 | 2,3-didehydro GA70 | 63.29 | 0.5 | 2 |
| Flos Carthami  (Honghua) | MOL001771 | poriferast-5-en-3beta-ol | 36.91 | 0.75 | 1 |
| Rhizoma Ligustici  (Chuanxiong) | MOL000433 | FA | 68.96 | 0.71 | 1 |
| Radix Clematidis  (Weilingxian) | MOL005603 | Heptyl phthalate | 42.26 | 0.31 | 1 |
| Semen Persicae  (Taoren) | MOL001361 | GA87 | 68.85 | 0.57 | 1 |
| Semen Persicae  (Taoren) | MOL001355 | GA63 | 65.54 | 0.54 | 1 |
| Semen Persicae  (Taoren) | MOL001351 | Gibberellin A44 | 101.61 | 0.54 | 1 |
| Semen Persicae  (Taoren) | MOL001349 | 4a-formyl-7alpha-hydroxy-1-methyl-8-methylidene-4aalpha,4bbeta-gibbane-1alpha,10beta-dicarboxylic acid | 88.6 | 0.46 | 1 |
| Semen Persicae  (Taoren) | MOL001340 | GA120 | 84.85 | 0.45 | 1 |

| Table S6 The binding energy of 8 core targets and corresponding ingredients | | |
| --- | --- | --- |
| Receptor protein | Small molecule ligand | Binding energy/ kcal.Mol |
| MYC | beta-carotene | -7.8 |
| TP53 | baicalein | -8.5 |
| PTGS2 | baicalein | -9.2 |
| PTGS2 | wallichilide | -7.7 |
| PTGS2 | 3-O-pcoumaroylquinicacid | -8.3 |
| PTGS2 | 6-Hydroxykaempferol | -9.3 |
| PTGS2 | betasitosterol | -9.3 |
| PTGS2 | campesterol | -9.2 |
| PTGS2 | hederagenin | -9.3 |
| PTGS2 | lignan | -7.6 |
| PTGS2 | Myricanone | -9.0 |
| PTGS2 | Perlolyrine | -9.0 |
| PTGS2 | quercetagetin | -9.7 |
| PTGS2 | Sitosterolalpha | -7.8 |
| PTGS2 | Stigmasterol | -8.0 |
| VEGFA | baicalein | -6.8 |
| VEGFA | beta-carotene | -7.9 |
| HIF-1Α | baicalein | -8.6 |
| CASP3 | baicalein | -7.5 |
| CASP3 | beta-carotene | -7.3 |
| CASP3 | beta-sitosterol | -7.2 |
| AKT1 | baicalein | -10.1 |
| AKT1 | beta-carotene | -9.7 |
| CTNNB1 | beta-carotene | -6.6 |
| TP53 | Atorvastatin | -8.6 |
| PTGS2 | Atorvastatin | -8.5 |
| VEGFA | Atorvastatin | -6.7 |
| HIF-1Α | Atorvastatin | -7.2 |
| CASP3 | Atorvastatin | -8.7 |
| AKT1 | Atorvastatin | -10.2 |
| MYC | Atorvastatin | -8.4 |

| Table S7 The UniProt accession numbers and links | | |
| --- | --- | --- |
| Core Targets | Accession Numbers | Links |
| MYC | P01106 | https://www.uniprot.org/uniprotkb/P01106/entry |
| TP53 | P04637 | https://www.uniprot.org/uniprotkb/P04637/entry |
| PTGS2 | P35354 | https://www.uniprot.org/uniprotkb/P35354/entry |
| VEGFA | P15692 | https://www.uniprot.org/uniprotkb/P15692/entry |
| HIF-1α | Q16665 | https://www.uniprot.org/uniprotkb/Q16665/entry |
| CASP3 | P42574 | https://www.uniprot.org/uniprotkb/P42574/entry |
| AKT1 | P31749 | https://www.uniprot.org/uniprotkb/P31749/entry |
| CTNNB1 | P35222 | https://www.uniprot.org/uniprotkb/P35222/entry |

| Table S8 The PubChem accession numbers and links | | |
| --- | --- | --- |
| Compounds | Accession Numbers | Links |
| Baicalein | 5281605 | <https://pubchem.ncbi.nlm.nih.gov/compound/5281605> |
| Beta-Carotene | 5280489 | <https://pubchem.ncbi.nlm.nih.gov/compound/5280489> |
| Myricanone | 161748 | <https://pubchem.ncbi.nlm.nih.gov/compound/161748> |
| Stigmasterol | 5280794 | <https://pubchem.ncbi.nlm.nih.gov/compound/5280794> |
| beta-sitosterol | 222284 | <https://pubchem.ncbi.nlm.nih.gov/compound/222284> |
| hederagenin | 73299 | <https://pubchem.ncbi.nlm.nih.gov/compound/73299> |
| 6-Hydroxykaempferol | 5281638 | https://pubchem.ncbi.nlm.nih.gov/compound/5281638 |
| Atorvastatin | 60823 | https://pubchem.ncbi.nlm.nih.gov/compound/60823 |
